# Unraveling the complex genetic landscape of *OTOF*-related hearing loss: a deep dive into cryptic variants and haplotype phasing

**DOI:** 10.1186/s10020-025-01225-2

**Published:** 2025-05-09

**Authors:** Pei-Hsuan Lin, Cheng-Yu Tsai, Yu-Ting Chiang, Chang-Han Ho, Yue-Sheng Lu, Jacob Shu-Jui Hsu, Yen-Fu Cheng, Shih-Feng Tsai, Chuan-Jen Hsu, Pei-Lung Chen, Chen-Chi Wu

**Affiliations:** 1 Graduate Institute of Clinical Medicine, College of Medicine, National Taiwan University, Taipei, 100229 Taiwan; 2 Department of Otolaryngology, National Taiwan University Hospital, Taipei, 100225 Taiwan; 3 Graduate Institute of Medical Genomics and Proteomics, College of Medicine, National Taiwan University, Taipei, 100233 Taiwan; 4 Department of Medical Research, Taipei Veterans General Hospital, Taipei, 112062 Taiwan; 5 Department of Otolaryngology-Head and Neck Surgery, Taipei Veterans General Hospital, Taipei, 112201 Taiwan; 6 Department of Otolaryngology-Head and Neck Surgery, School of Medicine, National Yang Ming Chiao Tung University, Taipei, 112304 Taiwan; 7 Institute of Molecular and Genomic Medicine, National Health Research Institute, Miaoli, 350401 Taiwan; 8 Department of Life Sciences and Institute of Genome Sciences, National Yang Ming Chiao Tung University, Taipei, 112304 Taiwan; 9 Department of Otolaryngology, Buddhist Tzuchi General Hospital, Taichung, 427003 Taiwan; 10 Institute of Molecular Medicine, National Taiwan University College of Medicine, Taipei, 100225 Taiwan; 11 Department of Medical Genetics, National Taiwan University Hospital, Taipei, 100226 Taiwan; 12 Department of Medical Research, National Taiwan University Hospital Hsin-Chu Branch, Hsinchu, 302041 Taiwan; 13 Department of Otolaryngology, National Taiwan University Hospital Hsin-Chu Branch, Hsinchu, 302041 Taiwan

**Keywords:** DFNB9, Short-read sequencing, Long-read sequencing, Splice prediction tool, Minigene assay

## Abstract

**Background:**

Pathogenic variants in *OTOF* are a major cause of auditory synaptopathy. However, challenges remain in interpreting *OTOF* variants, including difficulties in confirming haplotype phasing using traditional short-read sequencing (SRS) due to the large gene size, the potential incomplete penetrance of certain variants, and difficulties in assessing variants at non-canonical splice sites. This study aims to revisit the genetic landscape of *OTOF* variants in a Taiwanese non-syndromic auditory neuropathy spectrum disorder (ANSD) cohort using a combination of sequencing technologies, predictive tools, and experimental validations.

**Methods:**

We performed SRS to analyze *OTOF* variants in 65 unrelated Taiwanese patients diagnosed with non-syndromic ANSD, complemented by long-read sequencing (LRS) for haplotype phasing. A prediction-to-validation pipeline was implemented to assess the pathogenicity of cryptic variants using SpliceAI software and minigene assays.

**Results:**

Biallelic pathogenic *OTOF* variants were identified in 33 patients (50.8%), while monoallelic variants were found in five patients. Three novel variants, c.3864G > A (p.Ala1288 =), c.4501G > A (p.Ala1501Thr), and c.5813 + 2T > C, were detected. The pathogenicity of two non-canonical mis-splicing variants, c.3894 + 5G > C and c.3864G > A (p.Ala1288 =), was confirmed by minigene assays. LRS-based haplotype phasing revealed that the common missense variant c.5098G > C (p.Glu1700Gln) and the novel variant c.5975A > G (p.Lys1992Arg) are in *cis* and form a founder pathogenic allele in the Taiwanese population.

**Conclusions:**

Our study highlights the genetic heterogeneity of DFNB9 and emphasizes the importance of population-specific variant interpretation. The integration of advanced sequencing technologies, predictive algorithms, and functional validation assays will improve the accuracy of molecular diagnosis and inform personalized treatment strategies for individuals with DFNB9.

**Graphical Abstract:**

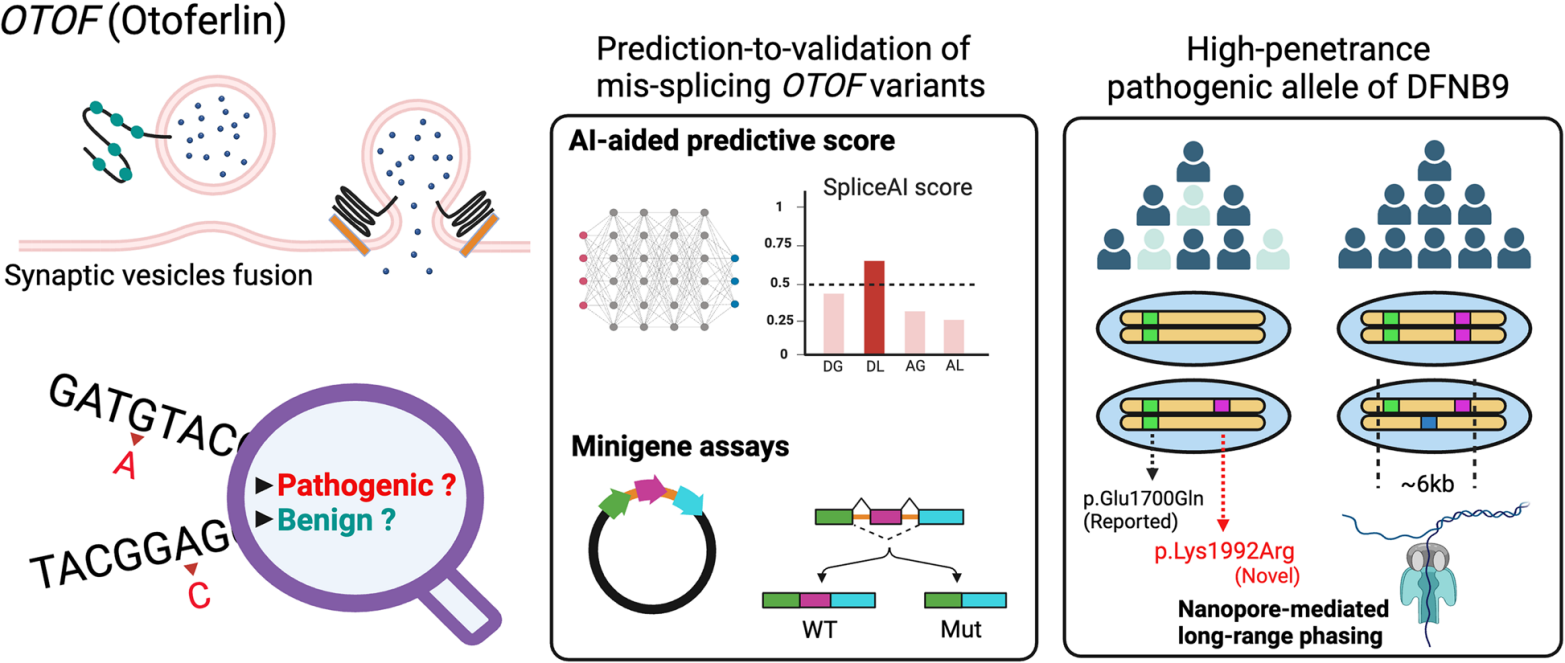

**Supplementary Information:**

The online version contains supplementary material available at 10.1186/s10020-025-01225-2.

## Introduction

Auditory neuropathy spectrum disorder (ANSD) encompasses a wide range of disease mechanisms, including synaptic encoding between inner hair cells and spiral ganglion neurons (i.e., auditory synaptopathy) and neural transmission of spiral ganglion neurons and beyond (i.e., auditory neuropathy) (Moser and Starr 2016). Pathogenic variants in the otoferlin gene (*OTOF*, OMIM #603681), which is crucial for synaptic vesicle trafficking in hair cells, are a major cause of auditory synaptopathy, also known as DFNB9 (Del Castillo and Del Castillo 2012; Wang et al. 2024; Iwasa et al. 2019; Azaiez et al. 2008). While the prevalence of *OTOF* variants varies in different populations, encouraging results from AAV-mediated gene therapy in both mouse models (Al-Moyed et al. 2019; Akil et al. 2019; Tang et al. 2023) and affected individuals (Lv et al. 2024; Qi et al. 2024) highlight its potential as a therapeutic strategy. However, the complex relationship between *OTOF* genotypes and clinical manifestations (phenotypes) poses a challenge in the management of DFNB9 patients (Ford et al. 2023).

More than 200 pathogenic and likely pathogenic variants have been identified (Vona et al. 2020), including several founder mutations in specific populations, such as p.Gln829Ter (Spanish) (Migliosi et al. 2002; Rodríguez-Ballesteros et al. 2008), p.Ala969LeufsTer30 (Argentinean) (Rodríguez-Ballesteros et al. 2008), p.Val1778Phe (Ashkenazi Jewish) (Fedick et al. 2016), p.Glu747Ter and p.Arg1792His (Saudi Arabian) (Almontashiri et al. 2018), and p.Arg1939Gln (Japanese and Korean) (Iwasa et al. 2013; Chang, et al. 2015). In addition, many DFNB9 patients have only one detectable pathogenic *OTOF* variant (monoallelic) instead of the two expected for a recessive disorder (Vona et al. 2020). This suggests that a significant number of patients either have a second undetected variant or a variant of uncertain significance (VUS) that has yet to be confirmed as contributing to their condition. This diagnostic uncertainty hinders the ability to determine the most appropriate treatment options.

Standard next-generation sequencing (NGS) approaches, constrained by short read lengths, often fail to resolve the phasing of distant variants within the large *OTOF* gene (> 100 kb), especially when parental samples are unavailable (Wojcik et al. 2023). Furthermore, determining the pathogenicity of cryptic variants at non-canonical splice sites requires a robust prediction-to-validation pipeline. In this study, we leverage long-read sequencing (LRS) technology, which is characterized by its ability to provide high-resolution haplotype phasing for distant variants (Wang et al. 2021; Oehler et al. 2023), to accurately determine the phase of clinically significant variants in DFNB9 patients. We also employ a comprehensive mis-splicing prediction-to-validation pipeline that integrates SpliceAI software (Jaganathan et al. 2019), a tool developed using deep neural network technology to predict the mutational effects on pre-mRNA splicing for any given variant, with minigene assays (Gaildrat et al. 2010) to functionally evaluate variants with unknown effects on the splicing process. This comprehensive approach aims to improve the accuracy of genetic diagnosis in DFNB9 and ultimately inform personalized treatment strategies.

## Materials and methods

### Subject recruitment and phenotype characterization

This study enrolled patients of Chinese Han ethnicity who were previously diagnosed with non-syndromic ANSD at the National Taiwan University Hospital (NTUH) between 2000 and 2023 (Table 1), some of whom were included in our previous publications (Lin et al. 2020; Lin et al. 2022; Chiu et al. 2010; Wu et al. 2019; Wu et al. 2018). ANSD was diagnosed on the basis of the presence of otoacoustic emissions and/or cochlear microphonics, together with absent or abnormal auditory brainstem responses in audiological examinations (Berlin et al. 2010). Basic demographic data, birth history, past medical history, and family history were collected for each patient. Audiological evaluations, including distortion product otoacoustic emissions, auditory brainstem response, auditory steady-state response, and behavioral audiometry, were performed according to age and neurological status. Non-contrast brain magnetic resonance imaging and high-resolution computed tomography of the temporal bone were performed to evaluate the inner ear structure, cochlear nerve, and central auditory pathway. Patients with known acquired risk factors (e.g., prematurity, kernicterus, perinatal insults), syndromic ANSD (e.g., autosomal dominant optic atrophy, Charcot-Marie-Tooth syndrome, Friedreich’s ataxia), or abnormal imaging findings (e.g., inner ear malformations, cochlear nerve aplasia/hypoplasia) were excluded. The study was approved by the NTUH Research Ethics Committee (201104025RC), and informed consents were obtained from all participants and/or their legal guardians.

**Table 1:** Diagnostic criteria for non-syndromic ANSD in this study

**Audiological findings**
• Presence of otoacoustic emissions and/or cochlear microphonics
• Absent or abnormal auditory brainstem responses
**Medical history**
• No evidence of acquired risk factors, such as prematurity, kernicterus, or perinatal insults
• No evidence of syndromic ANSD, such as autosomal dominant optic atrophy, Charcot-Marie-Tooth syndrome, or Friedreich’s ataxia
**Imaging studies**
• No evidence of inner ear malformations or cochlear nerve aplasia/hypoplasia

### Targeted short-read sequencing (SRS) and pathogenicity analysis of *OTOF* variants

Targeted SRS, based on conventional NGS procedures, was used as the initial diagnostic tool to investigate the genetic basis of non-syndromic ANSD in the study cohort. This approach utilized a targeted gene panel comprising 214 genes for hereditary hearing impairment, as previously reported (Lee et al. 2023). Using 300 bp paired-end reads on the Illumina MiSeq platform, sequencing and data processing followed established protocols detailed in our previous work (Lin et al. 2021). Variants with allele frequencies below the threshold of 1% in both reputable databases gnomAD (Karczewski et al. 2020) (ver. 2.1.1, last accessed August 10, 2024) and the community-specific Taiwan Biobank (Wei et al. 2021) (last accessed August 10, 2024) were prioritized for pathogenicity assessment. Variants classified as“pathogenic”or“likely pathogenic”based on the American College of Medical Genetics and Genomics (ACMG) guidelines (Richards et al. 2015; Oza et al. 2018) were considered causative variants for the phenotype.

Six prediction tools were used to assess the pathogenicity of missense variants: SIFT (Ng and Henikoff 2003), PolyPhen-2 (Adzhubei et al. 2013), MutationTaster (Schwarz et al. 2014), FATHMM-MKL (Shihab et al. 2015), CADD (Rentzsch et al. 2019), and DANN (Quang et al. 2014). Variant conservation analysis was performed using phyloP100way scores (Pollard et al. 2010), which incorporate multiple alignments of the human and 99 other vertebrate genomes to assess evolutionary conservation. SpliceAI (Jaganathan et al. 2019) was used to predict the potential presence of aberrant splicing variants. Variants with at least one delta score (scale: 0–1) for donor loss (DS-DL), donor gain (DS-DG), acceptor loss (DS-AL), or acceptor gain (DS-AG) greater than the recommended threshold (0.5) were selected for further experimental validation.

### Minigene assays

Minigene assays (Gaildrat et al. 2010) were performed to assess the effect of the identified variants on splicing. Genomic segments encompassing the variants of interest, including flanking exons and adjacent introns, were amplified and cloned into pcDNA3.1 plasmids using Gibson Assembly (Gibson et al. 2009) (Fig. S1). Wild-type constructs from a control individual with confirmed normal hearing were generated as negative controls. All the vectors have been confirmed to harbor their corresponding correct inserts using Sanger sequencing after clone construction.

These plasmids were transfected into HeLa cells in six-well plates using Lipofectamine at a density of approximately 5 × 10^5^ cells/well (Gaildrat et al. 2010). Twenty-four hours after transfection, cells were treated with 10 µg/mL puromycin to inhibit nonsense-mediated mRNA decay (Gaildrat et al. 2010). After 5.5 h, cells were harvested and RNA was extracted using Trizol reagent. RT-PCR primers were designed to target the outer edges of the flanking exons (Fig. S2A). Sanger sequencing was performed to confirm the presence of the inserted DNA constructs (Fig. S2B), and gel electrophoresis was used to assess the size of the cDNA products (Fig. S3). The resulting cDNA amplicons from both mutant and wild-type constructs were analyzed by SRS as described previously (Lin et al. 2021).

The Spliced Transcripts Alignment to a Reference (STAR) software (Dobin et al. 2013) was used to align cDNA reads to the *OTOF* exonic regions in the hg19 reference genome. The Sashimi plot function (Katz et al. 2015) within the Integrative Genomics Viewer (IGV) was used to visualize splice junctions in mutant RNA-seq data compared to the corresponding wild type.

### Long-read sequencing (LRS) and haplotype phasing

LRS assays for the *OTOF* variants c.5098G> C (p.Glu1700Gln) and c.5975A> G (p.Lys1992Arg) were performed using the Oxford Nanopore Technologies (ONT) MinION platform (Oxford, UK). Three cases (DE4777, DE4886, and DE5604) were selected for haplotype phasing (Fig. S4). A 6,657 bp region encompassing the variants of interest (hg19:chr2:26680442–26687098) was amplified by long-range PCR using TaKaRa LA Taq® kits (Cat. No. RR002A, Takara Bio Inc.) with specific primers: 5'-CGGATCAAAGACCGGTGCTATCTGC (forward) and 5'-AAAAGGAGGTGGGGTAGACAGGTGA (reverse). PCR products were purified using the Zymoclean™ Gel DNA Recovery Kit (Cat. No. D4008, Zymo Research Corp.).

The resulting raw electronic signal data from ONT were basecalled using Guppy (ver. 3.6) (Wick et al. 2019). Reads were filtered for quality (Q10) and length (> 6,500 bp) using Nanofilt (ver. 2.6) (De Coster et al. 2018). The filtered reads were then mapped to the hg19 reference genome using Minimap2 (ver. 2.17) (Li 2018), variants were called using DeepVariant (ver. 1.0) (Yun et al. 2020), and haplotype phasing was performed using WhatsHap (ver. 1.4) (Patterson et al. 2015) and Bamql (Masella et al. 2016).

## Results

### The *OTOF* variant spectrum identified in this study

In this study, 65 unrelated individuals with non-syndromic ANSD underwent targeted short-read sequencing (SRS) to identify potential pathogenic variants in the *OTOF* gene. Biallelic pathogenic variants (either homozygous or compound heterozygous) were identified in 33 patients (50.8%), whereas monoallelic variants were found in five patients (Table 2). A total of 18 different *OTOF* variants were detected in this cohort (Table 3), including three truncating variants, ten missense variants, three splice site variants, and two non-canonical splice site variants, c.3894 + 5G > C and c.3864G > A (p.Ala1288 =). No pathogenic variants in *PJVK* associated with DFNB59 hearing loss were identified.

**Table 2:** Summary of *OTOF* variants detected in this study

*OTOF* genotype (HGVSc)^a^	Amino acid change (HGVSp)^a^	No. of patients
**Biallelic variants**		***N* = 33**
c.[5098G > C];[5098G > C]	p.[Glu1700Gln];[Glu1700Gln]	12
c.[5098G > C];[2521G > A]	p.[Glu1700Gln];[Glu841Lys]	4
c.[5098G > C];[3704_3719del]	p.[Glu1700Gln];[Asp1235AlafsTer30]	2
c.[5098G > C];[4501G > A]	p.[Glu1700Gln];[Ala1501Thr]	2
c.[5098G > C];[1498C > T]	p.[Glu1700Gln];[Arg500Ter]	1
c.[5098G > C];[2279T > C]	p.[Glu1700Gln];[Leu760Pro]	1
c.[5098G > C];[3864G > A]	p.[Glu1700Gln];[Ala1288 =]	1
c.[5098G > C];[4030C > T]	p.[Glu1700Gln];[Arg1344Ter]	1
c.[5098G > C];[5000C > A]	p.[Glu1700Gln];[Ala1667Asp]	1
c.[5098G > C];[5197G > A]	p.[Glu1700Gln];[Glu1733Lys]	1
c.[5098G > C];[5203C > T]	p.[Glu1700Gln];[Arg1735Trp]	1
c.[5098G > C];[5335C > T]	p.[Glu1700Gln];[His1779Tyr]	1
c.[5098G > C];[5566C > T]	p.[Glu1700Gln];[Arg1856Trp]	1
c.[5098G > C];[3894 + 5G > C]	p.[Glu1700Gln];[?]	1
c.[5098G > C];[4023 + 1G > A]	p.[Glu1700Gln];[?]	1
c.[5098G > C];[4961-1G > A]	p.[Glu1700Gln];[?]	1
c.[5098G > C];[5813 + 2T > C]	p.[Glu1700Gln];[?]	1
**Monoallelic variants**		***N*** **= 5**
c.[5098G > C];[5098 =]	p.[Glu1700Gln];[Glu1700 =]	3
c.[4023 + 1G > A];[4023 + 1 =]	p.[?];[=]	1
c.[5287A > G];[5287 =]	p.[Arg1763Gly];[Arg1763 =]	1

^a^All variants are nomenclated according to the Human Genome Variation Society (HGVS) guidelines (http://varnomen.hgvs.org/), in terms of coding DNA (HGVSc) and protein (HGVSp) based on NM_001287489.2 and NP_001274418.1, respectively

**Table 3:** Detailed information on *OTOF* variants detected in this study

Patient	HGVS and loci of variants (NM_001287489.2)	Zygosity	Grpmax-AF^§^ (population)	TB-MAF*	Predictive scores	Database Assertions^#^	ACMG criteria^¥^ (classification)	Ref^€^	c.5975A > G (p.Lys1992Arg) (NM_001287489.2)
DE1117 (Reported)	c.5098G > C (p.Glu1700Gln) *hg19: chr2-26,686,837-C-G*	Homo	0.006774 (EAS)	0.007256	SIFT: 0.058 (T) PL-2: 0.898 (PD) CADD: 32	ClinVar: P DVD: P	PM3_VS, PP1_S, PP3, PP4 (Pathogenic)	Chiu et al. 2010; Wu et al. 2019)	Homo
DE1765 (Reported)	c.5098G > C (p.Glu1700Gln) *hg19: chr2-26,686,837-C-G*	Homo	0.006774 (EAS)	0.007256	SIFT: 0.058 (T) PL-2: 0.898 (PD) CADD: 32	ClinVar: P DVD: P	PM3_VS, PP1_S, PP3, PP4 (Pathogenic)	Chiu et al. 2010; Wu et al. 2019)	Homo
DE1780 (Reported)	c.5098G > C (p.Glu1700Gln) *hg19: chr2-26,686,837-C-G*	Homo	0.006774 (EAS)	0.007256	SIFT: 0.058 (T) PL-2: 0.898 (PD) CADD: 32	ClinVar: P DVD: P	PM3_VS, PP1_S, PP3, PP4 (Pathogenic)	Chiu et al. 2010; Wu et al. 2019)	Homo
DE1801 (Reported)	c.5098G > C (p.Glu1700Gln) *hg19: chr2-26,686,837-C-G*	Homo	0.006774 (EAS)	0.007256	SIFT: 0.058 (T) PL-2: 0.898 (PD) CADD: 32	ClinVar: P DVD: P	PM3_VS, PP1_S, PP3, PP4 (Pathogenic)	Chiu et al. 2010; Wu et al. 2019)	Homo
DE2401 (Reported)	c.5098G > C (p.Glu1700Gln) *hg19: chr2-26,686,837-C-G*	Homo	0.006774 (EAS)	0.007256	SIFT: 0.058 (T) PL-2: 0.898 (PD) CADD: 32	ClinVar: P DVD: P	PM3_VS, PP1_S, PP3, PP4 (Pathogenic)	Chiu et al. 2010; Wu et al. 2019)	Homo
DE3745 (Reported)	c.5098G > C p.Glu1700Gln) *hg19: chr2-26,686,837-C-G*	Homo	0.006774 (EAS)	0.007256	SIFT: 0.058 (T) PL-2: 0.898 (PD) CADD: 32	ClinVar: P DVD: P	PM3_VS, PP1_S, PP3, PP4 (Pathogenic)	Chiu et al. 2010; Wu et al. 2019)	Homo
DE4496 (Reported)	c.5098G > C (p.Glu1700Gln) *hg19: chr2-26,686,837-C-G*	Homo	0.006774 (EAS)	0.007256	SIFT: 0.058 (T) PL-2: 0.898 (PD) CADD: 32	ClinVar: P DVD: P	PM3_VS, PP1_S, PP3, PP4 (Pathogenic)	Chiu et al. 2010; Wu et al. 2019)	Homo
DE4856	c.5098G > C (p.Glu1700Gln) *hg19: chr2-26,686,837-C-G*	Homo	0.006774 (EAS)	0.007256	SIFT: 0.058 (T) PL-2: 0.898 (PD) CADD: 32	ClinVar: P DVD: P	PM3_VS, PP1_S, PP3, PP4 (Pathogenic)	Chiu et al. 2010; Wu et al. 2019)	Homo
DE7573	c.5098G > C (p.Glu1700Gln) *hg19: chr2-26,686,837-C-G*	Homo	0.006774 (EAS)	0.007256	SIFT: 0.058 (T) PL-2: 0.898 (PD) CADD: 32	ClinVar: P DVD: P	PM3_VS, PP1_S, PP3, PP4 (Pathogenic)	Chiu et al. 2010; Wu et al. 2019)	Homo
DE7942	c.5098G > C (p.Glu1700Gln) *hg19: chr2-26,686,837-C-G*	Homo	0.006774 (EAS)	0.007256	SIFT: 0.058 (T) PL-2: 0.898 (PD) CADD: 32	ClinVar: P DVD: P	PM3_VS, PP1_S, PP3, PP4 (Pathogenic)	Chiu et al. 2010; Wu et al. 2019)	Homo
A0826	c.5098G > C (p.Glu1700Gln) *hg19: chr2-26,686,837-C-G*	Homo	0.006774 (EAS)	0.007256	SIFT: 0.058 (T) PL-2: 0.898 (PD) CADD: 32	ClinVar: P DVD: P	PM3_VS, PP1_S, PP3, PP4 (Pathogenic)	Chiu et al. 2010; Wu et al. 2019)	Homo
A2421	c.5098G > C (p.Glu1700Gln) *hg19: chr2-26,686,837-C-G*	Homo	0.006774 (EAS)	0.007256	SIFT: 0.058 (T) PL-2: 0.898 (PD) CADD: 32	ClinVar: P DVD: P	PM3_VS, PP1_S, PP3, PP4 (Pathogenic)	Chiu et al. 2010; Wu et al. 2019)	Homo
DE2515	c.5098G > C (p.Glu1700Gln) *hg19: chr2-26,686,837-C-G*	Hetero	0.006774 (EAS)	0.007256	SIFT: 0.058 (T) PL-2: 0.898 (PD) CADD: 32	ClinVar: P DVD: P	PM3_VS, PP1_S, PP3, PP4 (Pathogenic)	Chiu et al. 2010; Wu et al. 2019)	Hetero
	c.2279T > C (p.Leu760Pro) *hg19: chr2-26,700,553-A-G*	Hetero	N.A	N.A	SIFT: 0.001 (D) PL-2: 0.956 (D) CADD: 27.3	ClinVar: N.A DVD: P	PM3_VS, PP1_S, PP3, PP4 (Pathogenic)	Wu et al. 2019)	
DE2575 (Reported)	c.5098G > C (p.Glu1700Gln) *hg19: chr2-26,686,837-C-G*	Hetero	0.006774 (EAS)	0.007256	SIFT: 0.058 (T) PL-2: 0.898 (PD) CADD: 32	ClinVar: P DVD: P	PM3_VS, PP1_S, PP3, PP4 (Pathogenic)	Chiu et al. 2010; Wu et al. 2019)	Hetero
	c.5000C > A (p.Ala1667Asp) *hg19: chr2-26,686,935-G-T*	Hetero	0.0001090 (EAS)	N.A	SIFT: 0.001 (D) PL-2: 0.732 (PD) CADD: 27.4	ClinVar: N.A DVD: VUS	PM2_P, PM3, PP3, PP4 (Likely Pathogenic)	Lin et al. 2022)	
DE3152 (Reported)	c.5098G > C (p.Glu1700Gln) *hg19: chr2-26,686,837-C-G*	Hetero	0.006774 (EAS)	0.007256	SIFT: 0.058 (T) PL-2: 0.898 (PD) CADD: 32	ClinVar: P DVD: P	PM3_VS, PP1_S, PP3, PP4 (Pathogenic)	Chiu et al. 2010; Wu et al. 2019)	Hetero
	c.2521G > A (p.Glu841Lys) *hg19: chr2-26,700,042-C-T*	Hetero	0.0002202 (EAS)	N.A	SIFT: 0.003 (D) PL-2: 0.522 (PD) CADD: 24.8	ClinVar: P/VUS DVD: P	PM2_P, PM3_VS, PP3, PP4 (Likely Pathogenic)	Wu et al. 2018; Kim et al. 2018)	
DE3247 (Reported)	c.5098G > C (p.Glu1700Gln) *hg19: chr2-26,686,837-C-G*	Hetero	0.006774 (EAS)	0.007256	SIFT: 0.058 (T) PL-2: 0.898 (PD) CADD: 32	ClinVar: P DVD: P	PM3_VS, PP1_S, PP3, PP4 (Pathogenic)	Chiu et al. 2010; Wu et al. 2019)	Hetero
	c.2521G > A (p.Glu841Lys) *hg19: chr2-26,700,042-C-T*	Hetero	0.0002202 (EAS)	N.A	SIFT: 0.003 (D) PL-2: 0.522 (PD) CADD: 24.8	ClinVar: P/VUS DVD: P	PM2_P, PM3_VS, PP3, PP4 (Likely Pathogenic)	Wu et al. 2018; Kim et al. 2018)	
DE3249 (Reported)	c.5098G > C (p.Glu1700Gln) *hg19: chr2-26,686,837-C-G*	Hetero	0.006774 (EAS)	0.007256	SIFT: 0.058 (T) PL-2: 0.898 (PD) CADD: 32	ClinVar: P DVD: P	PM3_VS, PP1_S, PP3, PP4 (Pathogenic)	Chiu et al. 2010; Wu et al. 2019)	Hetero
	c.4961-1G > A *hg19: chr2-26,686,975-C-T*	Hetero	N.A	N.A	SIFT: N.A PL-2: N.A CADD: 24.8	ClinVar: P/LP DVD: P	PVS1, PM2, PM3, PP4 (Pathogenic)	Wu et al. 2019; Wu et al. 2018)	
DE3532	c.5098G > C (p.Glu1700Gln) *hg19: chr2-26,686,837-C-G*	Hetero	0.006774 (EAS)	0.007256	SIFT: 0.058 (T) PL-2: 0.898 (PD) CADD: 32	ClinVar: P DVD: P	PM3_VS, PP1_S, PP3, PP4 (Pathogenic)	Chiu et al. 2010; Wu et al. 2019)	Hetero
	c.4023 + 1G > A *hg19: chr2-26,693,460-C-T*	Hetero	0.01178 (EAS)	0.015491	SIFT: N.A PL-2: N.A CADD: 26.3	ClinVar: VUS/LB DVD: B	PVS1, BA1, PM3, PP4 (VUS)	Wu et al. 2019; Jian et al. 2011)	
DE4417	c.5098G > C (p.Glu1700Gln) *hg19: chr2-26,686,837-C-G*	Hetero	0.006774 (EAS)	0.007256	SIFT: 0.058 (T) PL-2: 0.898 (PD) CADD: 32	ClinVar: P DVD: P	PM3_VS, PP1_S, PP3, PP4 (Pathogenic)	Chiu et al. 2010; Wu et al. 2019)	Hetero
	c.3894 + 5G > C *hg19: chr2-26,693,984-C-G*	Hetero	N.A	N.A	SIFT: N.A PL-2: N.A CADD: 18.3	ClinVar: N.A DVD: P	PVS1, PM2, PM3, PP4 (Pathogenic)	Wu et al. 2019)	
DE4618 (Reported)	c.5098G > C (p.Glu1700Gln) *hg19: chr2-26,686,837-C-G*	Hetero	0.006774 (EAS)	0.007256	SIFT: 0.058 (T) PL-2: 0.898 (PD) CADD: 32	ClinVar: P DVD: P	PM3_VS, PP1_S, PP3, PP4 (Pathogenic)	Chiu et al. 2010; Wu et al. 2019)	Hetero
	c.4030C > T (p.Arg1344Ter) *hg19: chr2-26,691,336-G-A*	Hetero	N.A	N.A	SIFT: N.A PL-2: N.A CADD: 46	ClinVar: P DVD: P	PVS1, PM2, PM3, PP4 (Pathogenic)	Wu et al. 2018; Baux et al. 2017)	
DE4732 (Reported)	c.5098G > C (p.Glu1700Gln) *hg19: chr2-26,686,837-C-G*	Hetero	0.006774 (EAS)	0.007256	SIFT: 0.058 (T) PL-2: 0.898 (PD) CADD: 32	ClinVar: P DVD: P	PM3_VS, PP1_S, PP3, PP4 (Pathogenic)	Chiu et al. 2010; Wu et al. 2019)	Hetero
	c.5566C > T (p.Arg1856Trp) *hg19: chr2-26,683,866-G-A*	Hetero	0.0001003 (EAS)	N.A	SIFT: 0.00 (D) PL-2: 0.960 (D) CADD: 25.1	ClinVar: P/VUS DVD: P	PM1, PM2_P, PM3, PM5, PP3, PP4 (Pathogenic)	Chang et al. 2015; Wu et al. 2019)	
DE4733	c.5098G > C (p.Glu1700Gln) *hg19: chr2-26,686,837-C-G*	Hetero	0.006774 (EAS)	0.007256	SIFT: 0.058 (T) PL-2: 0.898 (PD) CADD: 32	ClinVar: P DVD: P	PM3_VS, PP1_S, PP3, PP4 (Pathogenic)	Chiu et al. 2010; Wu et al. 2019)	Hetero
	c.2521G > A (p.Glu841Lys) *hg19: chr2-26,700,042-C-T*	Hetero	0.0002202 (EAS)	N.A	SIFT: 0.003 (D) PL-2: 0.522 (PD) CADD: 24.8	ClinVar: P/VUS DVD: P	PM2_P, PM3_VS, PP3, PP4 (Likely Pathogenic)	Wu et al. 2018; Kim et al. 2018)	
DE4777	c.5098G > C (p.Glu1700Gln) *hg19: chr2-26,686,837-C-G*	Hetero	0.006774 (EAS)	0.007256	SIFT: 0.058 (T) PL-2: 0.898 (PD) CADD: 32	ClinVar: P DVD: P	PM3_VS, PP1_S, PP3, PP4 (Pathogenic)	Chiu et al. 2010; Wu et al. 2019)	Hetero
	c.5197G > A (p.Glu1733Lys) *hg19: chr2-26,685,045-C-T*	Hetero	0.00005439 (EAS)	N.A	SIFT: 0.002 (D) PL-2: 0.998 (D) CADD: 35	ClinVar: P/LP DVD: P	PM1, PM2, PM3, PP3, PP4 (Pathogenic)	Wu et al. 2019; Choi et al. 2009)	
DE4886 (Reported)	c.5098G > C (p.Glu1700Gln) *hg19: chr2-26,686,837-C-G*	Hetero	0.006774 (EAS)	0.007256	SIFT: 0.058 (T) PL-2: 0.898 (PD) CADD: 32	ClinVar: P DVD: P	PM3_VS, PP1_S, PP3, PP4 (Pathogenic)	Chiu et al. 2010; Wu et al. 2019)	Hetero
	c.5203C > T (p.Arg1735Trp) *hg19: chr2-26,685,039-G-A*	Hetero	0.000008792 (EAS)	N.A	SIFT: 0.00 (D) PL-2: 1.000 (D) CADD: 34	ClinVar: LP DVD: P	PM1, PM2, PM3, PP3, PP4 (Likely Pathogenic)	Wu et al. 2019; Wu et al. 2018)	
DE4943 (Reported)	c.5098G > C (p.Glu1700Gln) *hg19: chr2-26,686,837-C-G*	Hetero	0.006774 (EAS)	0.007256	SIFT: 0.058 (T) PL-2: 0.898 (PD) CADD: 32	ClinVar: P DVD: P	PM3_VS, PP1_S, PP3, PP4 (Pathogenic)	Chiu et al. 2010; Wu et al. 2019)	Hetero
	c.1498C > T (p.Arg500Ter) *hg19: chr2-26,705,355-G-A*	Hetero	0.0001387 (OTH)	N.A	SIFT: N.A PL-2: N.A CADD: 40	ClinVar: P DVD: P	PVS1, PM2_P, PM3, PP4 (Pathogenic)	Wu et al. 2019; Wu et al. 2018)	
DE5486 (Reported)	c.5098G > C (p.Glu1700Gln) *hg19: chr2-26,686,837-C-G*	Hetero	0.006774 (EAS)	0.007256	SIFT: 0.058 (T) PL-2: 0.898 (PD) CADD: 32	ClinVar: P DVD: P	PM3_VS, PP1_S, PP3, PP4 (Pathogenic)	Chiu et al. 2010; Wu et al. 2019)	Hetero
	c.3704_3719del (p.Asp1235AlafsTer30) *hg19: chr2-26,696,014-CTGGGGGCCGAGCGGT-*	Hetero	N.A	N.A	SIFT: N.A PL-2: N.A CADD: N.A	ClinVar: N.A DVD: P	PVS1, PM2, PM3_S, PP4 (Pathogenic)	Wu et al. 2018; Zadro et al. 2010)	
DE5604	c.5098G > C (p.Glu1700Gln) *hg19: chr2-26,686,837-C-G*	Hetero	0.006774 (EAS)	0.007256	SIFT: 0.058 (T) PL-2: 0.898 (PD) CADD: 32	ClinVar: P DVD: P	PM3_VS, PP1_S, PP3, PP4 (Pathogenic)	Chiu et al. 2010; Wu et al. 2019)	Hetero
	c.5335C > T (p.His1779Tyr) *hg19: chr2-26,684,762-G-A*	Hetero	N.A	N.A	SIFT: 0.00 (D) PL-2: 0.661 (PD) CADD: 24.7	ClinVar: N.A DVD: P	PM1, PM2, PM3, PP3, PP4 (Likely Pathogenic)	Wu et al. 2019)	
DE6680 (Reported)	c.5098G > C (p.Glu1700Gln) *hg19: chr2-26,686,837-C-G*	Hetero	0.006774 (EAS)	0.007256	SIFT: 0.058 (T) PL-2: 0.898 (PD) CADD: 32	ClinVar: P DVD: P	PM3_VS, PP1_S, PP3, PP4 (Pathogenic)	Chiu et al. 2010; Wu et al. 2019)	Hetero
	c.3704_3719del (p.Asp1235AlafsTer30) *hg19: chr2-26,696,014-CTGGGGGCCGAGCGGT-*	Hetero	N.A	N.A	SIFT: N.A PL-2: N.A CADD: N.A	ClinVar: N.A DVD: P	PVS1, PM2, PM3_S, PP4 (Pathogenic)	Wu et al. 2018; Zadro et al. 2010)	
DE6695	c.5098G > C (p.Glu1700Gln) *hg19: chr2-26,686,837-C-G*	Hetero	0.006774 (EAS)	0.007256	SIFT: 0.058 (T) PL-2: 0.898 (PD) CADD: 32	ClinVar: P DVD: P	PM3_VS, PP1_S, PP3, PP4 (Pathogenic)	Chiu et al. 2010; Wu et al. 2019)	Hetero
	c.3864G > A (p.Ala1288 =) *hg19: chr2-26,695,387-C-T*	Hetero	N.A	N.A	SIFT: N.A PL-2: N.A CADD: 16.76	ClinVar: N.A DVD: N.A	PVS1, PM2, PP4 (Pathogenic)	This study	
A1387	c.5098G > C (p.Glu1700Gln) *hg19: chr2-26,686,837-C-G*	Hetero	0.006774 (EAS)	0.007256	SIFT: 0.058 (T) PL-2: 0.898 (PD) CADD: 32	ClinVar: P DVD: P	PM3_VS, PP1_S, PP3, PP4 (Pathogenic)	Chiu et al. 2010; Wu et al. 2019)	Hetero
	c.2521G > A (p.Glu841Lys) *hg19: chr2-26,700,042-C-T*	Hetero	0.0002202 (EAS)	N.A	SIFT: 0.003 (D) PL-2: 0.522 (PD) CADD: 24.8	ClinVar: P/VUS DVD: P	PM2_P, PM3_VS, PP3, PP4 (Likely Pathogenic)	Wu et al. 2018; Kim et al. 2018)	
A2693	c.5098G > C (p.Glu1700Gln) *hg19: chr2-26,686,837-C-G*	Hetero	0.006774 (EAS)	0.007256	SIFT: 0.058 (T) PL-2: 0.898 (PD) CADD: 32	ClinVar: P DVD: P	PM3_VS, PP1_S, PP3, PP4 (Pathogenic)	Chiu et al. 2010; Wu et al. 2019)	Hetero
	c.4501G > A (p.Ala1501Thr) *hg19: chr2-26,688,944-C-T*	Hetero	0.0002005 (EAS)	N.A	SIFT: 0.001 (D) PL-2: 0.670 (PD) CADD: 34	ClinVar: N.A DVD: VUS	PM2_P, PM3, PP3, PP4 (Likely Pathogenic)	This study	
A2776	c.5098G > C (p.Glu1700Gln) *hg19: chr2-26,686,837-C-G*	Hetero	0.006774 (EAS)	0.007256	SIFT: 0.058 (T) PL-2: 0.898 (PD) CADD: 32	ClinVar: P DVD: P	PM3_VS, PP1_S, PP3, PP4 (Pathogenic)	Chiu et al. 2010; Wu et al. 2019)	Hetero
	c.5813 + 2T > C *hg19: chr2-26,683,513-A-G*	Hetero	N.A	N.A	SIFT: N.A PL-2: N.A CADD: 24.3	ClinVar: N.A DVD: N.A	PVS1, PM2, PM3, PP4 (Pathogenic)	This study	
A3436	c.5098G > C (p.Glu1700Gln) *hg19: chr2-26,686,837-C-G*	Hetero	0.006774 (EAS)	0.007256	SIFT: 0.058 (T) PL-2: 0.898 (PD) CADD: 32	ClinVar: P DVD: P	PM3_VS, PP1_S, PP3, PP4 (Pathogenic)	Chiu et al. 2010; Wu et al. 2019)	Hetero
	c.4501G > A (p.Ala1501Thr) *hg19: chr2-26,688,944-C-T*	Hetero	0.0002005 (EAS)	N.A	SIFT: 0.001 (D) PL-2: 0.670 (PD) CADD: 34	ClinVar: N.A DVD: VUS	PM2_P, PM3, PP3, PP4 (Likely Pathogenic)	This study	
DE4636	c.4023 + 1G > A *hg19: chr2-26,693,460-C-T*	Hetero	0.01178 (EAS)	0.015491	SIFT: N.A PL-2: N.A CADD: 26.3	ClinVar: VUS/LB DVD: B	PVS1, BA1, PM3, PP4 (VUS)	Wu et al. 2019; Jian et al. 2011)	No
DE5555	c.5287A > G (p.Arg1763Gly) *hg19: chr2-26,684,955-T-C*	Hetero	N.A	N.A	SIFT: 0.002 (D) PL-2: 0.844 (PD) CADD: 25.1	ClinVar: N.A DVD: N.A	PM1, PM2, PP3, PP4 (Likely Pathogenic)	This study	No
DE6002	c.5098G > C (p.Glu1700Gln) *hg19: chr2-26,686,837-C-G*	Hetero	0.006774 (EAS)	0.007256	SIFT: 0.058 (T) PL-2: 0.898 (PD) CADD: 32	ClinVar: P DVD: P	PM3_VS, PP1_S, PP3, PP4 (Pathogenic)	Chiu et al. 2010; Wu et al. 2019)	Hetero
DE6441	c.5098G > C (p.Glu1700Gln) *hg19: chr2-26,686,837-C-G*	Hetero	0.006774 (EAS)	0.007256	SIFT: 0.058 (T) PL-2: 0.898 (PD) CADD: 32	ClinVar: P DVD: P	PM3_VS, PP1_S, PP3, PP4 (Pathogenic)	Chiu et al. 2010; Wu et al. 2019)	No
A1053	c.5098G > C (p.Glu1700Gln) *hg19: chr2-26,686,837-C-G*	Hetero	0.006774 (EAS)	0.007256	SIFT: 0.058 (T) PL-2: 0.898 (PD) CADD: 32	ClinVar: P DVD: P	PM3_VS, PP1_S, PP3, PP4 (Pathogenic)	Chiu et al. 2010; Wu et al. 2019)	Hetero

^§^Grpmax-AF: The maximum allele frequency across populations in the gnomAD database (Karczewski et al. 2020) (ver. 2.1.1, last accessed on August 10, 2024)

^*^TB-MAF: The minor allele frequency in the Taiwan Biobank (Wei et al. 2021), calculated from short-read sequencing data of 1517 healthy individuals (https://taiwanview.twbiobank.org.tw/browse38, accessed August 10, 2024). EAS: East Asian; OTH: Other

^¶^PL-2: PolyPhen-2 (HVAR). Abbreviations of assertion of SIFT and PolyPhen-2: D (damaging); PD (possibly damaging); T: (tolerant)

^#^Pathogenicity classifications were quoted from curated database of ClinVar (last accessed on November 29, 2024) (Landrum et al. 2016) and Deafness Variation Database (DVD, ver. 9) (Azaiez et al. 2018). P: pathogenic; LP: Likely pathogenic; VUS: Variant of uncertain significance; LB: likely benign; B: Benign

^¥^The criteria and variant classification of each variant were made according to the ClinGen expert-specified ACMG guidelines (Richards et al. 2015; Oza et al. 2018), where the content of c.5098G > C was quoted from the ClinGen expert-curated assertion (https://erepo.clinicalgenome.org/evrepo/ui/; CAID: CA345132; last accessed on November 29, 2024).VS: very strong; S: strong; P: supporting

^€^Publications for annotated variants were confirmed using ClinVar (Landrum et al. 2016), DVD (Azaiez et al. 2018), LOVD (Fokkema et al. 2005), and the variant2literature platform (Lin et al. 2019)

**Fig. 1:**
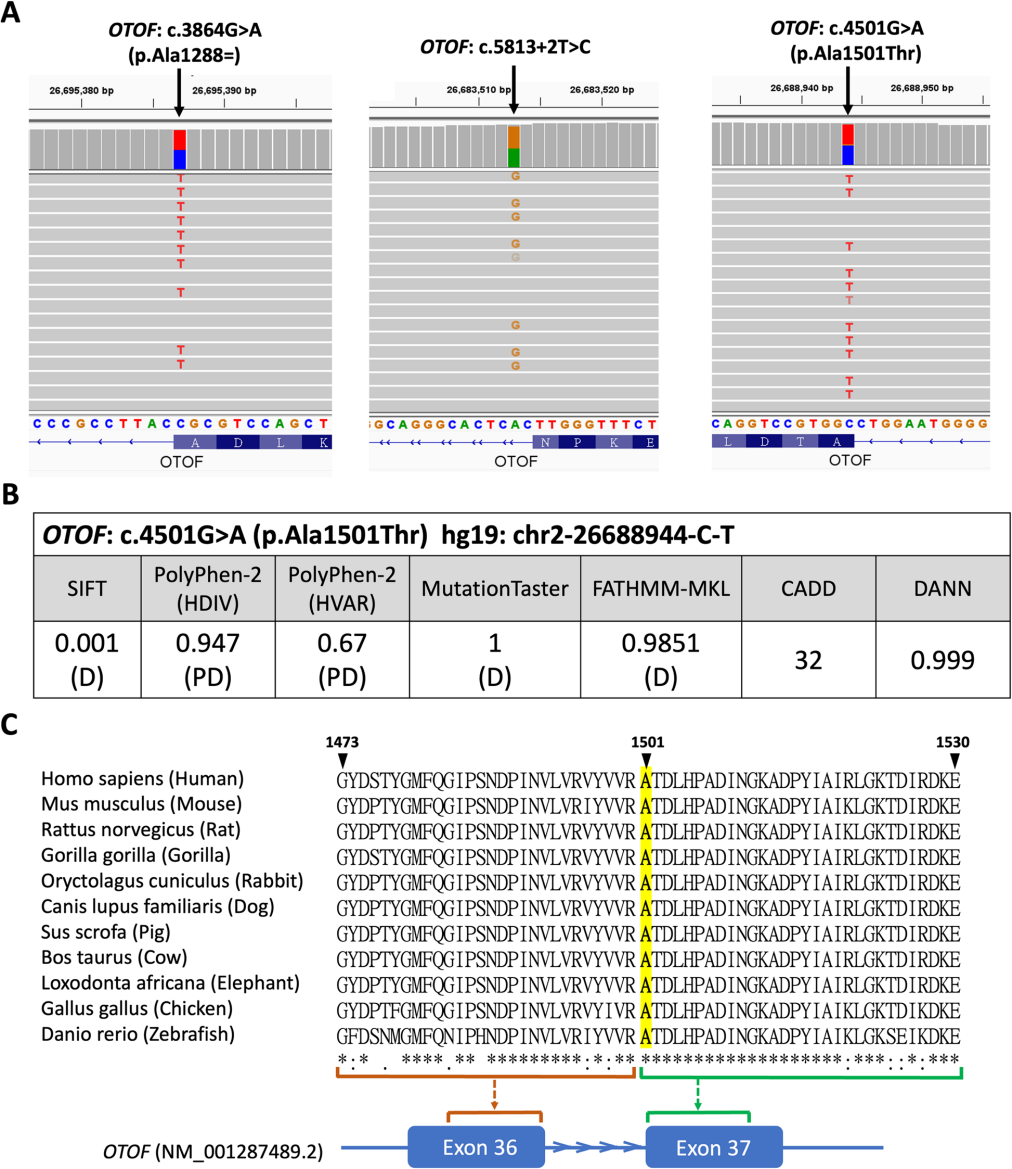
Overview of the three novel *OTOF* Variants. **A** Short-read sequencing (SRS) revealed all three novel variants, c.3864G > A (p.Ala1288 =), c.5813 + 2T > C, and c.4501G > A (p.Ala1501Thr), form compound heterozygosity with another pathogenic variant. **B** The missense variant c.4501G > A (p.Ala1501Thr) was classified as pathogenic based on multiple *in silico* prediction scores. **C** Multiple sequence alignment demonstrates the high conservation of the c.4501G > A (p.Ala1501Thr) coding region (located at the 5’ end of exon 37) across species. Homologous sequences for this alignment were obtained from the UCSC database (Table S1). Abbreviations: D (damaging); PD (possibly damaging)

Among these variants, three novel variants, c.3864G > A (p.Ala1288 =), c.4501G > A (p.Ala1501 Thr), and c.5813 + 2T > C, were discovered (Fig. 1A) in biallelic genotypes. The variant c.3864G > A (p.Ala1288 =), located at the last nucleotide (3’ end) of exon 30 near the 5’ splice donor site, was initially classified as “benign” by ACMG criteria due to its predicted synonymous consequence (p.Ala1288 =). However, SpliceAI (Jaganathan, et al. 2019) predicted that this variant was likely to disrupt the splicing process (DS-DL = 0.61). The splice site variant c.5813 + 2T > C, located at the splice donor site, is confidently classified as pathogenic due to the expected disruption of splicing. The missense variant c.4501G > A (p.Ala1501Thr) is also considered pathogenic based on multiple predictive scores (Fig. 1B) and its high conservation across species (Fig. 1C).

### Prediction and validation of cryptic *OTOF* variants leading to aberrant splicing

A combined approach using predictive tools and experimental validation improves the detection of cryptic variants that cause aberrant splicing. To evaluate the impact of such variants, especially those located at non-canonical splice sites, we implemented a prediction-to-validation pipeline integrating SpliceAI software and minigene assays. In addition to the aforementioned c.3864G > A (p.Ala1288 =) variant, another reported variant in the DFNB9, c.3894 + 5G > C (Wu et al. 2019), was also subjected to SpliceAI prediction for potential aberrant splicing. High SpliceAI scores (> 0.5), indicating a possible mis-splicing consequence, were observed for both variants (Table S2).

**Fig. 2:**
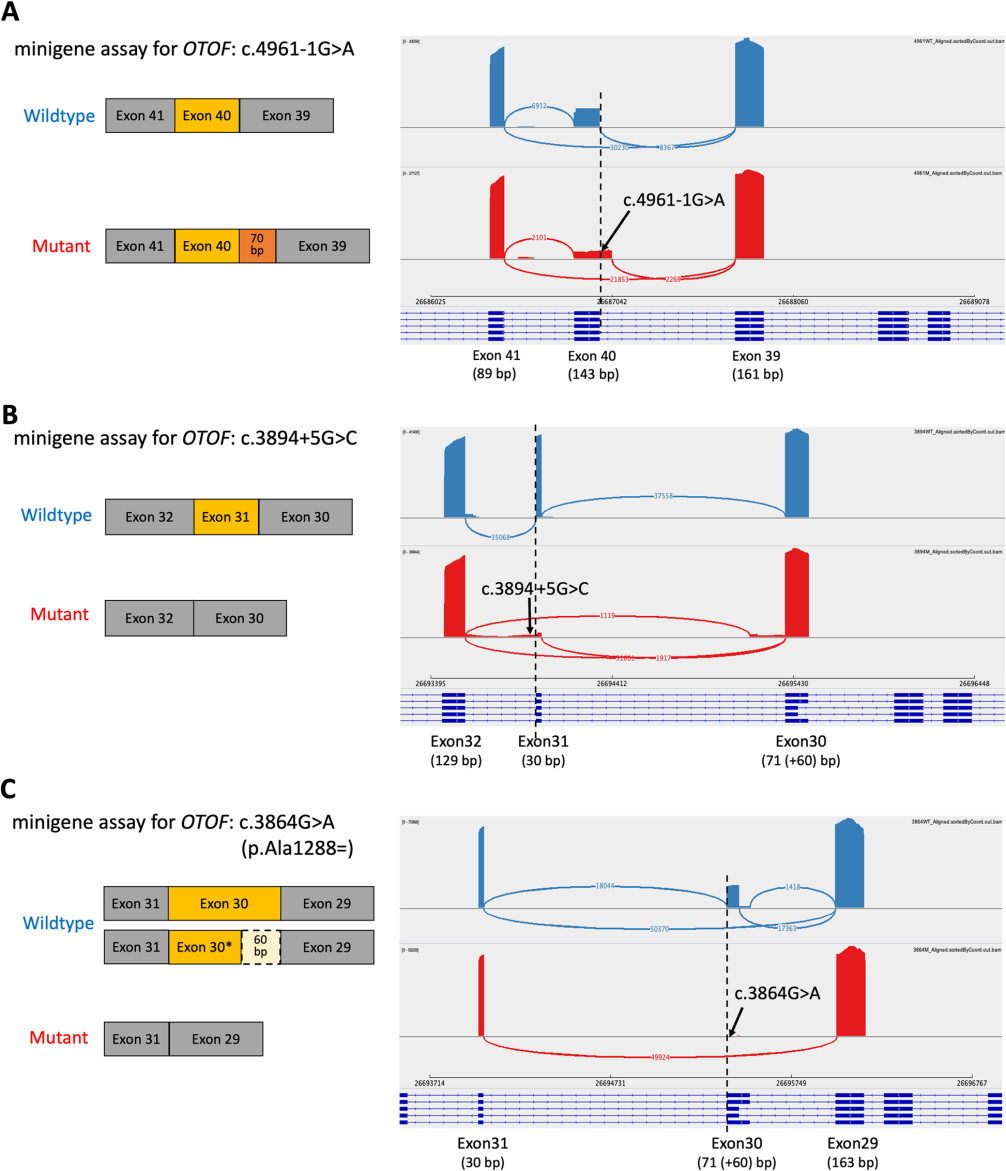
Differential expression of cDNA products in minigene assays. Schematic representation of aberrant splicing events and corresponding mapping plots of RT-PCR products for *OTOF* variants: (**A**) c.4961-1G > A, (**B**) c.3894 + 5G > C, and (**C**) c.3864G > A (p.Ala1288 =)

Subsequent validation using minigene assays was conducted to assess their mutational effects. The canonical splice variant c.4961-1G > A, which had a high SpliceAI score for acceptor loss (DS-AL = 0.96), served as a positive control. The minigene assay confirmed aberrant splicing for this variant, resulting in an extended exon 40 segment in the mutant transcript (Fig. 2A and Fig. S3A). This extension led to a 70 bp out-of-frame insertion from the 3’ end of intron 39 (chr2:26686975–26687044) and a premature stop codon in the encoded protein (Table S3), demonstrating the feasibility of this experimental validation method.

Minigene assays were then performed on the two non-canonical splice site variants. The results showed that c.3894 + 5G > C caused exon 31 skipping in the mutant cDNA products (Fig. 2B and Fig. S3B), resulting in an in-frame deletion (30 bp) in the RNA spliced transcript and a loss of 10 amino acids in the encoded protein (Table S3). For the c.3864G > A (p.Ala1288 =) variant, differential expression of cDNA products was observed (Fig. 2C). The wild type expressed two cDNA isoforms with different exon 30 lengths (exon 30 and exon 30*) (Fig. S3C), consistent with the reported multiple otoferlin isoforms (long and short forms) co-expressed in the cochlear tissue (Yasunaga et al. 2000). In contrast, the c.3864G > A (p.Ala1288 =) mutant expressed only the exon 30-skipped form, leading to a 131 bp out-of-frame deletion in the RNA transcript and premature termination in the encoded protein (Table S3), suggesting that this variant disrupts the normal splicing process of *OTOF*.

### Revisiting the penetrance of c.5098G > C (p.Glu1700Gln) and the novel variant c.5975A > G (p.Lys1992Arg)

Among the identified pathogenic *OTOF* variants, the missense variant, c.5098G > C (p.Glu1700Gln) (hg19:chr2:26686837-C-G), located in exon 40 of transcript NM_001287489.2, is prevalent in the Taiwanese DFNB9 patients (Chiu et al. 2010; Wu et al. 2019). This variant was predominantly found in either the homozygous or heterozygous state in our cohort (Table 2). However, our recent newborn screening using automated auditory brainstem responses identified four individuals with normal hearing but segregating two c.5098G > C (p.Glu1700Gln) alleles (Fig. S5), which were detected by additional newborn genetic screening selected by their parents. Subsequent follow-up assessments, including behavioral audiometry, distortion product otoacoustic emissions, and diagnostic auditory brainstem responses, consistently demonstrated normal hearing. This, together with the existence of a control individual carrying homozygous c.5098G > C (p.Glu1700Gln) curated in gnomAD (*n* = 1 in 9977 East Asian individuals, ver. 2.1.1), prompted a reassessment of its penetrance for the observed auditory synaptopathy symptoms.

**Fig. 3:**
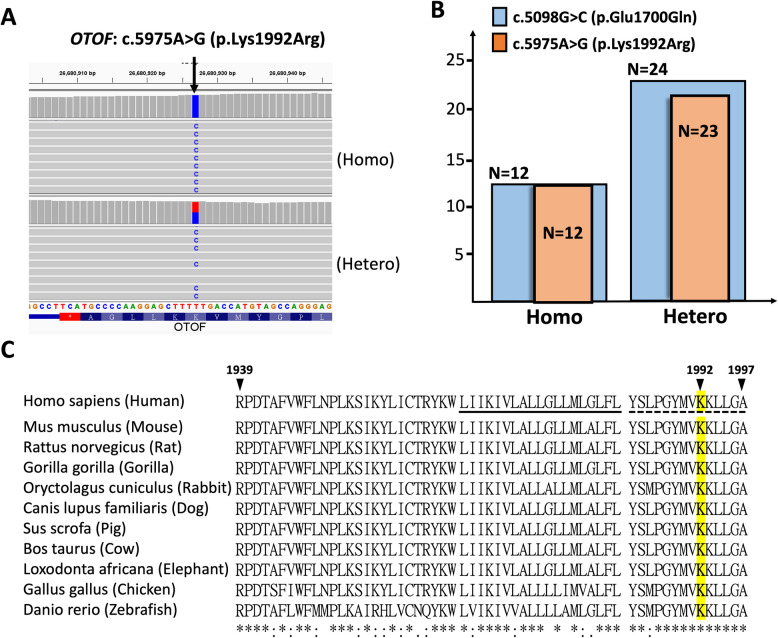
Overview of the novel *OTOF* founder variant c.5975A > G (p.Lys1992Arg). **A** Representative short-read sequencing (SRS) results of the c.5975A > G (p.Lys1992Arg) variant in homozygous and heterozygous individuals among DFNB9 patients. **B** A high co-occurrence rate between the c.5098G > C (p.Glu1700Gln) and c.5975A > G (p.Lys1992Arg) variants, in homozygous and heterozygous individuals, is observed in our DFNB9 patients. **C** Cross-species alignment of homologous sequences shows the high conservation of the coding region of NM_001287489.2 exon 46 (residues 1939 to 1997). The c.5975A > G (p.Lys1992Arg) variant in human otoferlin is located within this conserved C-terminal loop (dashed underline), adjacent to the reported transmembrane domain (solid underline). Homologous sequences were obtained from the UCSC database (Table S1)

Interestingly, c.5098G > C (p.Glu1700Gln) frequently co-occurred with another missense variant, c.5975A > G (p.Lys1992Arg) (NM_001287489.2: exon 46, hg19:chr2:26680927-T-C), in our cohort (Fig. 3A). All 12 homozygous c.5098G > C (p.Glu1700Gln) cases also carried two copies of the c.5975A > G (p.Lys1992Arg) variant, and 23 of 24 heterozygous c.5098G > C (p.Glu1700Gln) cases also carried the c.5975A > G (p.Lys1992Arg) (Table 3 & Fig. 3B). Human *OTOF*- and mouse *Otof*-encoded otoferlin have been reported to have multiple spliced transcripts with differential expression in the brain and cochlear tissues (Yasunaga et al. 2000). The c.5975A > G (p.Lys1992Arg) variant is located within exon 46 of the NM_001287489.2 mRNA transcript, the predominant otoferlin isoform expressed in the cochlea (Tang et al. 2023; Vona et al. 2020; Varga et al. 2003). This variant affects a highly conserved residue within the C-terminal loop of otoferlin (Fig. 3C; Table S1), a region known to harbor pathogenic variants associated with hearing loss (Varga et al. 2003; Rodríguez-Ballesteros et al. 2003). These findings suggest a potential association between c.5975A > G (p.Lys1992Arg) and DFNB9.

A comparison of the pathogenicity profiles for c.5975A > G (p.Lys1992Arg) and c.5098G > C (p.Glu1700Gln) is shown in Table 4. Notably, c.5975A > G (p.Lys1992Arg) has lower allele frequencies in both gnomAD (0.0008018 vs. 0.0068) and the Taiwan Biobank (0.002 vs. 0.0073), indicating its relative rarity. In addition, c.5975A > G (p.Lys1992Arg) shows higher evolutionary conservation as reflected by the PhyloP100way scores (7.871 vs. 7.619). Both c.5975A > G (p.Lys1992Arg) and c.5098G > C (p.Glu1700Gln) received similar pathogenicity predictions from several in silico tools. The collective evidence suggests that c.5975A > G (p.Lys1992Arg) may contribute to the DFNB9 disease phenotype in patients harboring the c.5098G > C (p.Glu1700Gln) variant. Given the pathogenic assertion of c.5098G > C (p.Glu1700Gln) and corresponding ACMG rules by the ClinGen expert panel (CAID: CA345132, mentioned in Table 3), we recommend an ACMG classification of “Pathogenic” for c.5975A > G (p.Lys1992Arg) with the additive PM2_Supporting rule (low Grpmax frequency). Further evidence is needed to definitively clarify the pathogenic role of c.5975A > G (p.Lys1992Arg).

**Table 4:** Comparison between *OTOF* c.5975A > G (p.Lys1992Arg) and c.5098G > C (p.Glu1700Gln)

	c.5975A > G (p.Lys1992Arg)	c.5098G > C (p.Glu1700Gln)
**Allele frequencies**		
Grpmax-AF (gnomAD)^a^	0.0008018 (16/19954)	0.0068 (135/19930)
Individuals with homozygote^b^	0/9977 (EAS)	1/9965 (EAS)
TB-MAF (Taiwan Biobank)^c^	0.002 (6/1513)	0.0073 (22/1516)
**Conservation prediction**		
phyloP100way	7.871	7.619
**Pathogenicity prediction**		
SIFT	0.346 (T)	0.058 (T)
PolyPhen-2-HDIV	1 (D)	0.996 (D)
PolyPhen-2-HVAR	0.997 (D)	0.898 (PD)
MutationTaster	1 (D)	1 (D)
FATHMM-MKL	0.98321 (D)	0.98954 (D)
CADD	21.6	32
DANN	0.999	0.998
**Pathogenicity assertions**^**d**^	PM2_P, PM3_VS, PP1_S, PP3, PP4 【Pathogenic】 (This study)	PM3_VS, PP1_S, PP3, PP4 【Pathogenic】 (By ClinGen)

*Abbreviations*: *D* Damaging, *T* Tolerant

^a^Last accessed on gnomAD (ver. 2.1.1) on August 10, 2024. (allele number in parentheses)

^b^The number of homozygotes in the selected subpopulation with the maximal allele frequency (Grpmax-AF) in gnomAD (ver. 2.1.1). EAS: East Asian

^c^Last accessed at Taiwan Biobank on August 10, 2024. (allele number in parentheses)

^d^The criteria and variant classification of each variant were made according to the ClinGen expert-specified ACMG guidelines (Richards et al. 2015; Oza et al. 2018). VS: very strong; S: strong; P: supporting

### Haplotype phasing for c.5975A > G (p.Lys1992Arg) and c.5098G > C (p.Glu1700Gln)

We then conducted haplotype phasing to determine the *cis* or *trans* configuration between c.5975A > G (p.Lys1992Arg) and c.5098G > C (p.Glu1700Gln). Due to the significant distance between these variants (~ 5.9 kb), long-range PCR combined with LRS on the ONT platform was used. Three cases (DE4777, DE4886, DE5604) harboring pathogenic heterozygous variants c.5197G > A (p.Glu1733Lys), c.5203C > T (p.Arg1735Trp), and c.5335C > T (p.His1779Tyr), respectively, located between c.5975A > G (p.Lys1992Arg) and c.5098G > C (p.Glu1700Gln), which could serve as markers of haplotype phasing, were selected for LRS (Fig. S4A). PCR primers were designed to generate ~ 6.6 kb amplicons (Fig. S4B) encompassing both c.5098G > C (p.Glu1700Gln) and c.5975 A > G (p.Lys1992Arg) in these samples.

**Fig. 4:**
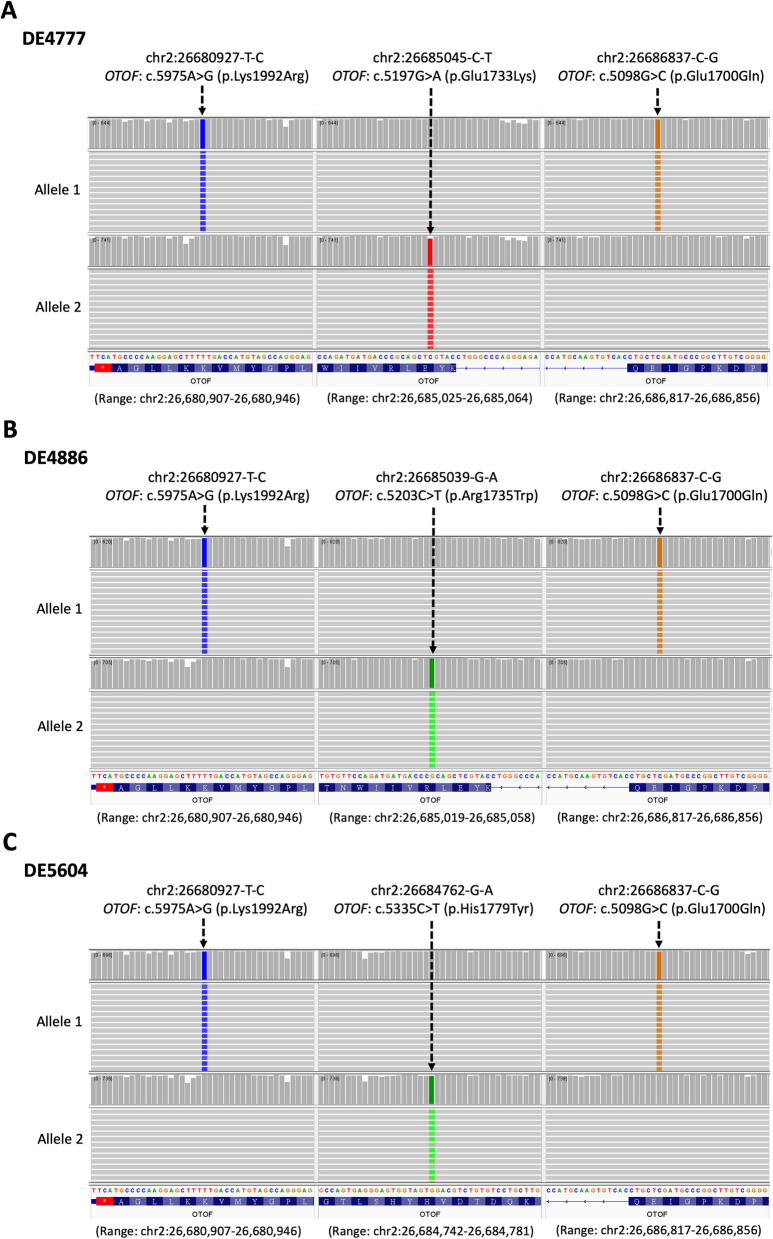
Haplotype phasing of pathogenic alleles containing the c.5098G > C (p.Glu1700Gln) and c.5975A > G (p.Lys1992Arg) variants. Long-read sequencing (LRS) was performed on the Oxford Nanopore Technologies (ONT) platform for samples (**A**) DE4777, (**B**) DE4886, and (**C**) DE5604. Phased haplotypes, designated as allele 1 and allele 2, were determined based on the presence of c.5098G > C (p.Glu1700Gln) (right) and the respective compound heterozygous variants (middle)

LRS analysis confirmed that, in all three cases, c.5098G > C (p.Glu1700Gln) was *in* *trans* with the other pathogenic variants, as previously established. Furthermore, c.5975A > G (p.Lys1992Arg) was located on the same haplotype as c.5098G > C (p.Glu1700Gln) (Fig. 4). The phasing tool WhatsHap (Patterson et al. 2015) was used to independently validate these findings (Fig. S6).

Taken together, these results demonstrate that c.5098G > C (p.Glu1700Gln) and c.5975A > G (p.Lys1992Arg) exist in a *cis* configuration on one allele, while the other pathogenic variants are located on the opposite allele, resulting in compound heterozygosity within *OTOF* and leading to DFNB9.

## Discussion

In this study, we utilized a combination of advanced sequencing, bioinformatics, and experimental tools to investigate the *OTOF* gene in non-syndromic ANSD patients. Our results confirm that pathogenic *OTOF* variants are the predominant genetic cause of this disorder in the Taiwanese population, identifying biallelic variants in 33 patients and monoallelic variants in five patients. Notably, we used SpliceAI and minigene assays to identify and validate the pathogenicity of two cryptic variants, c.3894 + 5G> C and c.3864G> A (p.Ala1288 =), that lead to aberrant splicing. Furthermore, LRS-based haplotype phasing revealed that the *cis* configuration of c.5098G> C (p.Glu1700Gln) and c.5975A> G (p.Lys1992Arg) constitutes a distinct pathogenic allele that, when *in* *trans* with other pathogenic variants, can result in compound heterozygosity in *OTOF* and lead to DFNB9.

Our integrative sequencing and analysis approach achieved a high diagnostic yield (approximately 50.8%) of pathogenic *OTOF* variants in non-syndromic ANSD patients comparable to previous studies (Zhang et al. 2016; Wang et al. 2010; Matsunaga et al. 2012). While pathogenic *OTOF* variants are a well-established major cause of this disorder, the prevalence of specific variants and the overall prevalence of *OTOF*-associated hearing loss exhibit significant inter-population variability. *OTOF* variants have been identified as the cause of hearing impairment in approximately 5% of Turkish patients (Duman et al. 2011), 3% of Pakistani patients (Choi et al. 2009; Richard et al. 2019), 2.4% of European-American patients (Sloan-Heggen et al. 2016), 1.9% of French patients (Baux et al. 2017), and 1.7% of Japanese patients (Iwasa et al. 2019) who were not pre-selected for ANSD. Genetic epidemiologic studies in patients specifically with ANSD have tended to be smaller and have yielded inconsistent results. For example, one study screening the *OTOF* gene in 37 Chinese patients with congenital ANSD had a diagnostic yield of 41.2% (Zhang et al. 2016), while another study in 73 Chinese Han patients with ANSD identified *OTOF* variants in only 5.5% of patients (Wang et al. 2010). Our study highlights the importance of considering population-specific genetic landscapes and implementing advanced sequencing and analytic tools in the diagnosis and management of non-syndromic ANSD.

Assessing the pathogenicity of cryptic variants, which disrupt normal splicing and can lead to genetic disorders, is more challenging than assessing null or missense variants due to the limited availability of prediction tools. To overcome this, we developed a prediction-to-validation pipeline that integrates SpliceAI (Jaganathan et al. 2019), a mis-splicing prediction tool, with minigene assays (Gaildrat et al. 2010). This approach successfully identified two cryptic variants, c.3894 + 5G > C and c.3864G > A (p.Ala1288 =), with deleterious effects on splicing. Notably, this pipeline detected the pathogenicity of the synonymous variant c.3864G > A (p.Ala1288 =), which was initially classified as benign by the ACMG guidelines (Richards et al. 2015) but was subsequently confirmed to cause complete skipping of exon 30. This demonstrates the power of our pipeline to detect mis-splicing variants, including those at non-canonical splice sites, that may be missed by traditional ACMG-based analysis. This observation underscores the clinical significance of our research in the non-syndromic ANSD cohort. It is important to note that higher SpliceAI scores indicate a higher probability of aberrant splicing, not necessarily the severity of the effect on gene products. We recommend confirming these effects through experimental validation, such as minigene assays, rather than relying solely on prediction results. While SpliceAI has outperformed many other prediction tools (Wai et al. 2020; Riepe et al. 2021; Jang et al. 2022; Smith and Kitzman 2023), it is advisable to incorporate multiple prediction tools or weighted scores that integrate multiple predictions (Rowlands et al. 2021). This approach could increase the sensitivity and robustness of predictions and effectively guide subsequent experimental validation in clinical genetics research.

Our previous research identified c.5098G> C (p.Glu1700Gln) as the most common pathogenic *OTOF* variant in the Taiwanese non-syndromic ANSD cohort (Chiu et al. 2010). This variant has also been reported in other Han Chinese patients but is rarely observed elsewhere (Qiu et al. 2019; Chen et al. 2018; Thorpe et al. 2022). However, its penetrance became questionable when we recently identified four unaffected individuals homozygous for c.5098G> C (p.Glu1700Gln) through newborn screening. Meanwhile, we have identified another variant, c.5975A> G (p.Lys1992Arg), which frequently co-occurs with c.5098G> C (p.Glu1700Gln) in our DFNB9 patients. LRS-mediated haplotype phasing revealed that these two variants are in linkage disequilibrium. The c.5975A> G (p.Lys1992Arg) showed a low allelic frequency in East Asian and Taiwanese populations, high evolutionary conservation and pathogenicity scores similar to c.5098G> C (p.Glu1700Gln), suggesting a potentially deleterious effect (Karczewski et al. 2020; Wei et al. 2021). Notably, c.5975A> G (p.Lys1992Arg) homozygotes were absent from normal populations and the aforementioned four unaffected newborns homozygous for c.5098G> C (p.Glu1700Gln) (Fig. S5). Given the reported clinical variability of p.Glu1700Gln (Ford et al. 2023), the frequent co-occurrence of c.5098G> C (p.Glu1700Gln) with c.5975A> G (p.Lys1992Arg) on the haplotype identified in our study suggests a potential influence of this co-occurrence on the observed phenotype. The *cis* configuration of c.5098G> C (p.Glu1700Gln) and c.5975A> G (p.Lys1992Arg) appears to represent a distinct pathogenic allele that, when *in* *trans* with the other pathogenic variants, can result in compound heterozygosity in *OTOF* and lead to DFNB9. The pathogenic role of c.5975A> G (p.Lys1992Arg) requires further clarification with more functional evidence or clinical reports with atypical clinical presentation of c.5098G> C (p.Glu1700Gln). Given the expert-based assertions and available evidence shown in this study, there are three scenarios to be verified in the future: (1) c.5098G> C (p.Glu1700Gln) is still the pathogenic variant with reduced penetrance or an atypical presentation. (2) c.5098G> C (p.Glu1700Gln) and c.5975A> G (p.Lys1992Arg) together form a pathogenic haplotype that, when *in trans* with other pathogenic variants, contributes to the disease phenotype. (3) c.5975A> G (p.Lys1992Arg) is the pathogenic variant, and previous reports supporting the role of c.5098G> C (p.Glu1700Gln) are all due to its linkage disequilibrium with c.5975A> G (p.Lys1992Arg).

Clarifying the pathogenicity of missense variants and identifying pathogenic cryptic variants are essential for accurate molecular diagnosis and personalized treatment of DFNB9 patients. This is particularly relevant in light of ongoing clinical trials for DFNB9 gene therapy. While current gene augmentation strategies may have limited long-term efficacy, gene editing approaches offer a promising avenue for more durable gene correction (Yan et al. 2023). Therefore, understanding the pathogenicity of specific variants is critical to improving diagnostic accuracy and informing the development of effective and durable treatment options for DFNB9.

Importantly, our comprehensive approach integrating multiple sequencing, analytical and experimental tools can be extended to other forms of autosomal recessive hearing loss. Non-confirmatory diagnoses with monoallelic pathogenic variants are not uncommon in genetic testing for conditions such as *GJB2*-related hearing loss (Lin et al. 2021). Even in patients with phenotypes that have been strongly linked to a specific gene, such as the association between enlarged vestibular aqueduct (EVA) and *SLC26A4*, a significant proportion of EVA patients have only one or no detectable pathogenic variants (Honda and Griffith 2022; Smits et al. 2022). It has been hypothesized that cryptic variants may exist in the non-coding regions of *GJB2* (Tang et al. 2006; Mani et al. 2009) and *SLC26A4* (Lin et al. 2019; Chen et al. 2011; Yuan et al. 2012), but this remains to be verified. A thorough and systematic investigation of cryptic variants will become increasingly important, especially in the post-genomic era of precision medicine as gene therapy for hereditary hearing loss becomes more accessible.

The strength of this study lies in the integrative approach combining LRS-based haplotype phasing and minigene assays to elucidate the genetic landscape of DFNB9. However, several limitations should be acknowledged. First, our SRS and LRS did not cover the entire genome and may have missed pathogenic variants in deep introns and regulatory elements of the *OTOF* gene. Second, due to the infeasibility of obtaining patient cochlear tissue, our minigene assays were performed in vitro using a cell line platform rather than tissue-specific models. Future advances, such as reduced sequencing costs enabling whole-genome LRS and the development of inner ear organoid platforms, may enhance the ability to study pathogenic variants in *OTOF*, especially those with monoallelic variants, or in other deafness genes in a more physiologically relevant context.

## Conclusion

In conclusion, our study highlights the genetic heterogeneity of DFNB9 and emphasizes the importance of population-specific variant interpretation. By integrating advanced sequencing technologies, predictive algorithms, and functional validation assays, we identified novel pathogenic variants and clarified the pathogenicity of previously reported variants. Our findings have significant implications for accurate molecular diagnosis, genetic counseling, and the development of personalized therapeutic strategies for individuals with DFNB9.

## Supplementary Information

Supplementary Material 1.

## Data Availability

No datasets were generated or analysed during the current study.
