# Supplementary material for: Unraveling the complex genetic landscape of OTOF-related hearing loss: a deep dive into cryptic variants and haplotype phasing
Source: Mol Med. 2025 May 9;31:181. doi: 10.1186/s10020-025-01225-2 (PMC12063237; doi:10.1186/s10020-025-01225-2)
Supplement: Supplementary file 1 — Supplementary Material 1. [file 10020_2025_1225_MOESM1_ESM.pdf]

Supplementary Materials

Unraveling the complex genetic landscape of *OTOF*-related hearing loss: a deep dive into cryptic variants and haplotype phasing

*Pei-Hsuan Lin [1,2 #], Cheng-Yu Tsai [2,3 #], Yu-Ting Chiang [2,3], Chang-Han Ho [2], Yue-Sheng Lu [2,3], Jacob Shu-Jui Hsu [3], Yen-Fu Cheng [4,5,6], Shih-Feng Tsai [7,8], Chuan-Jen Hsu [2,9], Pei-Lung Chen [1,3,10,11] *, and Chen-Chi Wu [1,2,12,13] **

1) Graduate Institute of Clinical Medicine, College of Medicine, National Taiwan University, Taipei, 100229, Taiwan.

2) Department of Otolaryngology, National Taiwan University Hospital, Taipei, 100225, Taiwan.

3) Graduate Institute of Medical Genomics and Proteomics, College of Medicine, National Taiwan University, Taipei, 100233, Taiwan.

4) Department of Medical Research, Taipei Veterans General Hospital, Taipei, 112062, Taiwan.

5) Department of Otolaryngology-Head and Neck Surgery, Taipei Veterans General Hospital, Taipei, 112201, Taiwan.

6) Department of Otolaryngology-Head and Neck Surgery, School of Medicine, National Yang Ming Chiao Tung University, Taipei, 112304, Taiwan.

7) Institute of Molecular and Genomic Medicine, National Health Research Institute, Miaoli, 350401, Taiwan.

8) Department of Life Sciences and Institute of Genome Sciences, National Yang Ming Chiao Tung University, Taipei, 112304, Taiwan

9) Department of Otolaryngology, Buddhist Tzuchi General Hospital, Taichung, 427003, Taiwan

10) Institute of Molecular Medicine, National Taiwan University College of Medicine, Taipei, 100225, Taiwan

11) Department of Medical Genetics, National Taiwan University Hospital, Taipei, 100226, Taiwan

12) Department of Medical Research, National Taiwan University Hospital Hsin-Chu Branch, Hsinchu, 302041, Taiwan

13) Department of Otolaryngology, National Taiwan University Hospital Hsin-Chu Branch, Hsinchu, 302041, Taiwan

# These authors contributed equally: Pei-Hsuan Lin (P.H.L.) and Cheng-Yu Tsai (C.Y.T.)

* **Correspondence**: Pei-Lung Chen (P.L.C.) and Chen-Chi Wu (C.C.W.)

- Graduate Institute of Medical Genomics and Proteomics, National Taiwan University College of Medicine, No.1 Jen-Ai Road Sec.1, 100233 Taipei, Taiwan (P.L.C.) paylong@ntu.edu.tw (P.L.C.)
- Department of Otolaryngology, National Taiwan University Hospital, No.7 Chung-Shan South Rd., 100225 Taipei, Taiwan (C.C.W.) chenchiwu@ntuh.gov.tw (C.C.W.)

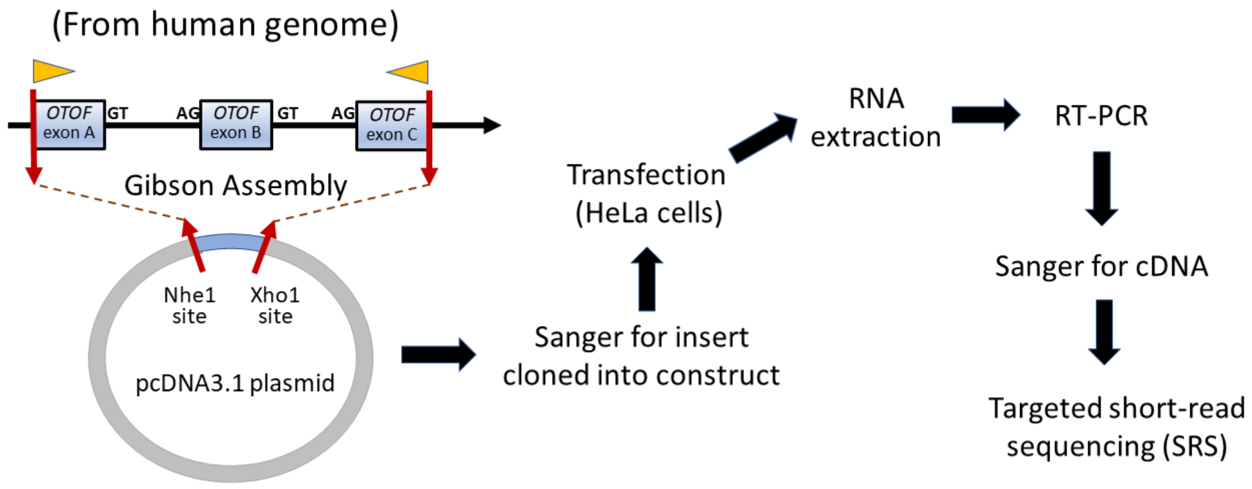

Figure S1. Illustration of the minigene construction for targeted inserts with or without targeted variants, cloned into the plasmid using restriction enzymes. All the inserts are derived from human genomes, with the segments used for mutant clones obtained directly from patient samples by the PCR amplification.

A

Variant	Strand	Primer sequences for RT-PCR
c.4961-1G>A	Forward	5'-AAGCTGGACATGGCTACAAT
	Reverse	5'-TTCCGAGGTGAGATGTCC
c.3894+5G>A	Forward	5'-AAGCTGGTCAGGCTTCTC
	Reverse	5'-GTCAATGGAGGCAAAGTACT
c.3864G>A (p.Ala1288=)	Forward	5'-GACCTCCCAGAGAACGAGC
	Reverse	5'-CACATCCACCTTGACAACAGC

B

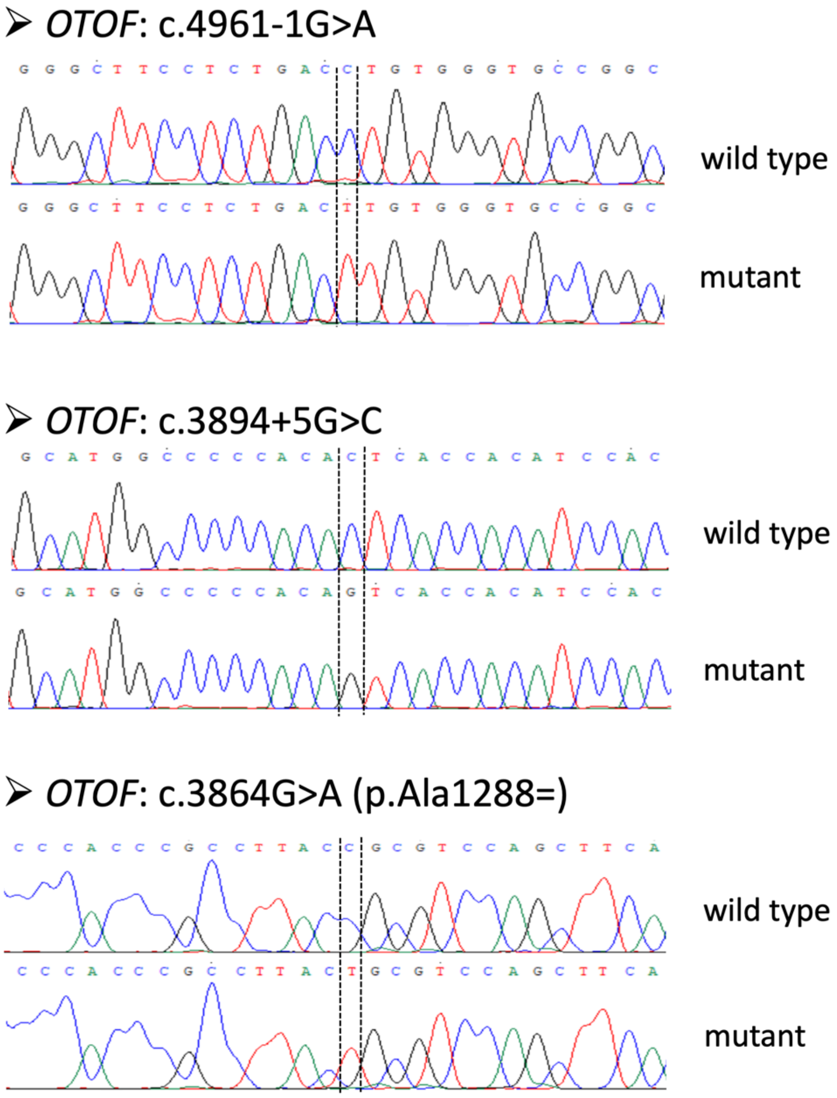

Figure S2. Overview of RT-PCR primers and products for minigene assays. (A) RT-PCR primers targeting at the outer edges of exon within the DNA insert constructs. (B) Sanger sequencing of the DNA insert constructs, comparing variants (mutants) with their respective wild types.

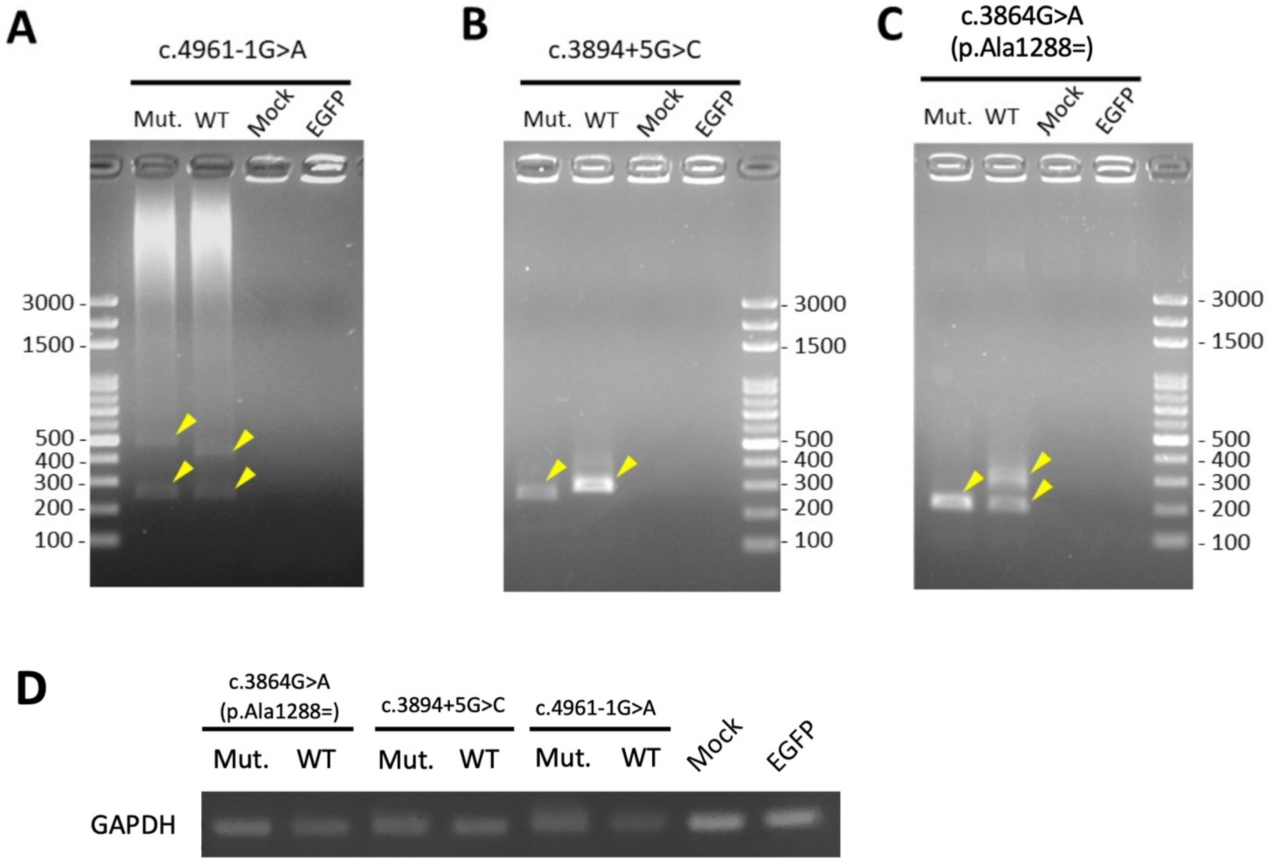

Figure S3. Gel electrophoresis of RT-PCR products for minigene assays. (A) c.4961-1G>A; (B) c.3894+5G>C; (C) c.3864G>A (p.Ala1288=). (D) GAPDH detection for all samples. Mock: control cell line without transfections; EGFP: "enhanced green fluorescent protein," the reporter gene of the plasmid without any targeted inserts; GAPDH: "glyceraldehyde 3-phosphate dehydrogenase," the housekeeping gene of the cell lines. Yellow arrows: the predominant bands of cDNA amplicons after RT-PCR for analyses of short-read sequencings.

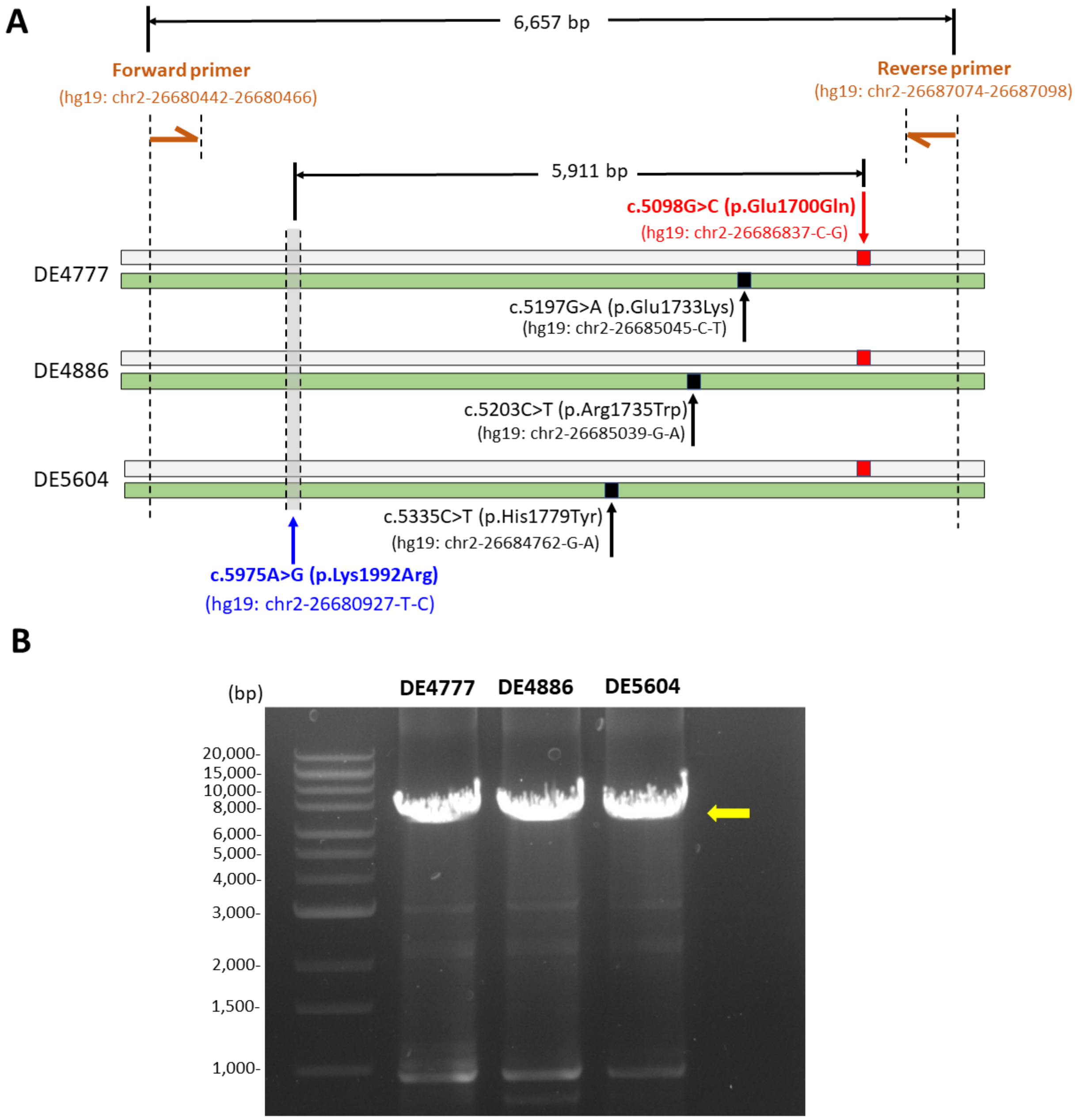

Figure S4. Long-range amplification for haplotype phasing of *OTOF* variants c.5975A>G (p.Lys1992Arg) and c.5098G>C (p.Glu1700Gln). (A) Illustration of the primers designed to generate 6,657 bp amplicons covering c.5975A>G (p.Lys1992Arg) (blue) and c.5098G>C (p.Glu1700Gln) (red) and their compound heterozygous variants (black) in samples DE4777, DE4886 and DE5604. (B) Long-range PCR products with expected lengths from the three samples (yellow arrow) for subsequent long-read sequencing.

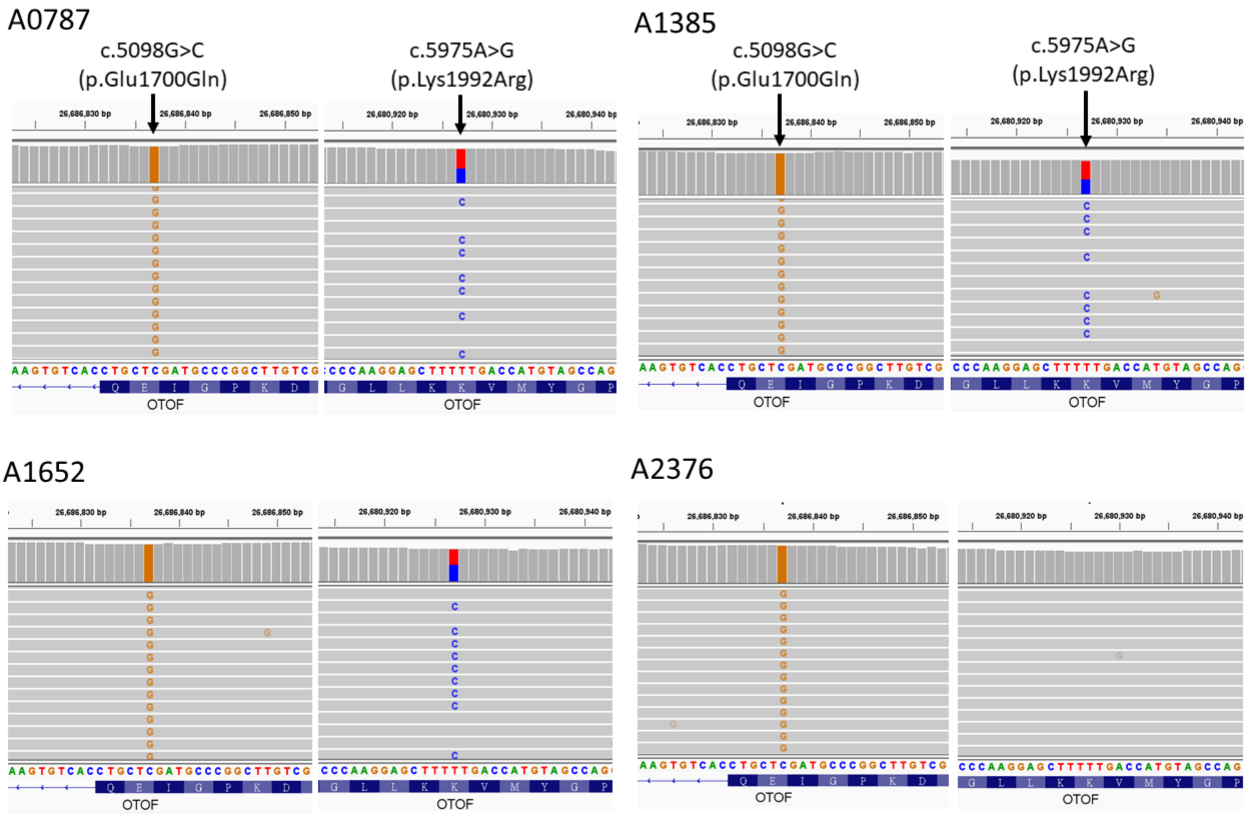

Figure S5. Genotypes of *OTOF* variants in four normal hearing newborns homozygous for the c.5098G>C (p.Glu1700Gln) allele, with either one or zero copies of the c.5975A>G (p.Lys1992Arg) allele.

DE4777

#CHROM	POS	ID	REF	ALT	QUAL	FILTER	INFO	FORMAT	default
chr2	26680927	.	T	C	57.6	PASS	.	GT:GQ:DP:AD:VAF:PL:PS	1|0:43:589:324,254:0.431239:57,0,43:26680927
chr2	26685045	.	C	T	66.4	PASS	.	GT:GQ:DP:AD:VAF:PL:PS	0|1:63:503:221,257:0.510934:66,0,65:26680927
chr2	26686837	.	C	G	52.1	PASS	.	GT:GQ:DP:AD:VAF:PL:PS	1|0:51:614:281,315:0.513029:52,0,55:26680927

DE4886

#CHROM	POS	ID	REF	ALT	QUAL	FILTER	INFO	FORMAT	default
chr2	26680927	.	T	C	54.7	PASS	.	GT:GQ:DP:AD:VAF:PL:PS	1|0:37:488:266,210:0.430328:54,0,36:26680927
chr2	26685039	.	G	A	10.5	PASS	.	GT:GQ:DP:AD:VAF:PL:PS	0|1:10:355:151,173:0.487324:10,0,23:26680927
chr2	26686837	.	C	G	48.8	PASS	.	GT:GQ:DP:AD:VAF:PL:PS	1|0:37:485:204,273:0.562887:48,0,36:26680927

DE5604

#CHROM	POS	ID	REF	ALT	QUAL	FILTER	INFO	FORMAT	default
chr2	26680927	.	T	C	57.1	PASS	.	GT:GQ:DP:AD:VAF:PL:PS	1|0:39:629:331,280:0.445151:57,0,38:26680927
chr2	26684762	.	G	A	55.9	PASS	.	GT:GQ:DP:AD:VAF:PL:PS	0|1:51:670:363,301:0.449254:55,0,53:26680927
chr2	26686837	.	C	G	50.8	PASS	.	GT:GQ:DP:AD:VAF:PL:PS	1|0:50:641:291,332:0.517941:50,0,55:26680927

Figure S6. Summary of phasing results using WhatsHap software. Abbreviations: GT: genotype; VAF: variant allele frequencies; PS: phasing (chr2-26680927 was selected as the basic benchmark for phasing in this analysis).

Table S1. Genomic regions from different species used for multiple sequence alignment (MSA) of *OTOF* coding sequences on c.4501G>A (p.Ala1501Thr) and c.5975A>G (p.Lys1992Arg) variants

Species (reference genome)	MSA of c.4501G>A (p.Ala1501Thr)	MSA of c.5975A>G (p.Lys1992Arg)
Human (hg19)	chr2:26688855-26689671	chr2-26680911-26681087
Mouse (mm39)	chr5:30533253-30533707	chr5-30525207-30525383
Rat (rn7)	chr6:26015097-26015551	chr6-26023643-26023819
Gorilla (gorGor6)	chr2A:42609405-42610219	chr2A-42601478-42601654
Rabbit (oryCun2)	chr2:159471035-159471365	chr2-159477312-159477488
Dog (canFam6)	chr17:20421563-20422277	chr17-20414093-20414269
Pig (susScr11)	chr3:112572854-112573338	chr3-112579919-112580095
Cow (bosTau9)	chr11:73085102-73085575	chr11-73093556-73093732
Elephant (loxAfr3)	scaffold_20:39797432-39797868	scaffold_20-39789878-39790054
Chicken (galGal6)	chr3:105328274-105329527	chr3-105335765-105335941
Zebrafish (danRer11)	chr17:50335952-50337139	chr17-50320287-50320463

Table S2. SpliceAI prediction scores for the three variants investigated in the minigene assays

Patients	HGVS of variants (NM_001287489.2)	Locus of variants (hg19)	DS-AL	DS-AG	DS-DL	DS-DG
DE3249	c.4961-1G>A	*chr2-26686975-C-T*	**0.96**	0.33	0.02	0.01
DE6695	c.3864G>A (p.Ala1288=)	*chr2-26695387-C-T*	0.2	0	**0.61**	0.23
DE4417	c.3894+5G>C	*chr2-26693984-C-G*	0.42	0	**0.86**	0.03

Note: DS (delta scores) are the predicted scores from SpliceAI (scale: 0-1). Scores in bold exceed the recommended criterion (0.5).

Abbreviations: AL: Acceptor Loss; AG: Acceptor Gain; DL: Donor Loss; DG: Donor Gain.

Table S3. The summary of resultant RNA-level and protein-level effects in the minigene assays

Patients	Mutation types	RNA-level aberrant spliced effects (NM_001287489.2)	Protein-level expected effects (NP_001274418.1)
DE3249	Out-of-frame insertion	r.4960_4961insucccuagucccagcaaaggucuucugguuc ugcugagccaugugugcagcugagccgccggcacccacag (70bp)	p.(Gly1654Valfs*18)
DE6695	In-frame deletion	r.3865_3894del (30bp)	p.(Thr1289_Val1298del)
DE4417	Out-of-frame deletion	r.3734_3864del (131bp)	p.(Val1245Aspfs*3)

All the descriptions of aberrant RNA and protein effects are normalized by HGVS nomenclature checker Mutalyzer (ver3, https://mutalyzer.nl/).
